# (Quasi) multitask support vector regression with heuristic hyperparameter optimization for whole-genome prediction of complex traits: a case study with carcass traits in broilers

**DOI:** 10.1093/g3journal/jkad109

**Published:** 2023-05-22

**Authors:** Anderson Antonio Carvalho Alves, Arthur Francisco Araujo Fernandes, Fernando Brito Lopes, Vivian Breen, Rachel Hawken, Daniel Gianola, Guilherme Jordão de Magalhães Rosa

**Affiliations:** Department of Animal and Dairy Sciences, University of Wisconsin-Madison, Madison, WI 53706, USA; Cobb-Vantress Inc., Siloam Springs, AR 72761, USA; Cobb-Vantress Inc., Siloam Springs, AR 72761, USA; Cobb-Vantress Inc., Siloam Springs, AR 72761, USA; Cobb-Vantress Inc., Siloam Springs, AR 72761, USA; Department of Animal and Dairy Sciences, University of Wisconsin-Madison, Madison, WI 53706, USA; Department of Animal and Dairy Sciences, University of Wisconsin-Madison, Madison, WI 53706, USA

**Keywords:** genetic algorithm, Genomic Prediction, machine learning, multitrait models, kernel methods, GenPred, Shared Data Resource

## Abstract

This study investigates nonlinear kernels for multitrait (MT) genomic prediction using support vector regression (SVR) models. We assessed the predictive ability delivered by single-trait (ST) and MT models for 2 carcass traits (*CT1* and *CT2*) measured in purebred broiler chickens. The MT models also included information on indicator traits measured in vivo [*Growth* and feed efficiency trait (*FE*)]. We proposed an approach termed (quasi) multitask SVR (QMTSVR), with hyperparameter optimization performed via genetic algorithm. ST and MT Bayesian shrinkage and variable selection models [genomic best linear unbiased predictor (GBLUP), BayesC (BC), and reproducing kernel Hilbert space (RKHS) regression] were employed as benchmarks. MT models were trained using 2 validation designs (CV1 and CV2), which differ if the information on secondary traits is available in the testing set. Models’ predictive ability was assessed with prediction accuracy (ACC; i.e. the correlation between predicted and observed values, divided by the square root of phenotype accuracy), standardized root-mean-squared error (RMSE*), and inflation factor (*b*). To account for potential bias in CV2-style predictions, we also computed a parametric estimate of accuracy ($\mathrm{AC}C_{\mathrm{par}}$). Predictive ability metrics varied according to trait, model, and validation design (CV1 or CV2), ranging from 0.71 to 0.84 for ACC, 0.78 to 0.92 for RMSE*, and between 0.82 and 1.34 for *b*. The highest ACC and smallest RMSE* were achieved with QMTSVR-CV2 in both traits. We observed that for *CT1*, model/validation design selection was sensitive to the choice of accuracy metric (ACC or ACC_par_). Nonetheless, the higher predictive accuracy of QMTSVR over MTGBLUP and MTBC was replicated across accuracy metrics, besides the similar performance between the proposed method and the MTRKHS model. Results showed that the proposed approach is competitive with conventional MT Bayesian regression models using either Gaussian or spike–slab multivariate priors.

## Introduction

Genomic selection (GS) is a suitable alternative to the traditional pedigree-based genetic evaluation, generally providing higher prediction accuracy for young animals and contributing to faster genetic progress in different livestock species (Mrode *et al*. 2019). In animal breeding programs, many quantitative traits are sex-specific, laborious, time-consuming, or expensive to measure. Hence, strategies that allow rapid, accurate, and resource-efficient predictions are of particular interest to fully exploit the potential of genome-enabled selection. In this context, the interest in multitrait genomic prediction (MT-GP) for aggregating information from correlated traits has largely increased in the last few years (VanRaden and Sullivan 2010; Calus and Veerkamp 2011; Guo *et al*. 2014; Karaman *et al*. 2018; Gianola and Fernando 2020; Manzanilla-Pech *et al*. 2020), as it can potentially increase the prediction accuracy of complex traits, especially when the training sample size is small (Manzanilla-Pech *et al*. 2020).

MT-GP is especially interesting for carcass traits as they most often require slaughtering the animals for assessing their phenotypic performance. Exploring the information of indicator traits measured on live animals is expected to benefit the genomic evaluation of postmortem traits. Nonetheless, implementation of MT-GP models can be challenging in practical applications, as such methods are computationally intensive and require knowledge of covariance components that may be hard to estimate when many traits are analyzed jointly. Additionally, most MT-GP models assume only additive effects of genetic markers, while ignoring potential nonlinear effects.

Alternatively, various machine learning (ML) methods have been viewed as capable of dealing with complex high-dimensional data from modern genotyping technologies (Nayeri *et al*. 2019). To date, most ML applications in genomic prediction use only the information available for a single trait at a time, while few efforts have been devoted to considering information from multiple outputs, a problem also known as multitask learning (MTL) in the ML literature. Early proposals of unifying MT-GP and MTL perspectives include He *et al*. (2016), who used a cluster-based MTL algorithm for predicting fruit and seed quality traits, providing competitive predictive performance relative to Bayesian MT-GP models. Similarly, Montesinos-López *et al*. (2018) compared the performance of Bayesian multitrait genomic best linear unbiased predictor (MTGBLUP) and multitrait deep learning (MT-DL) models for genomic prediction of several traits of maize and wheat lines in different environmental conditions. The authors noticed that MT-DL was superior to the MTGBLUP only when covariates corresponding to genotype by environment interaction were not considered in the model.

A popular class of ML procedures applied to whole-genome prediction is the so-called kernel-based regression methods, e.g. reproducing kernel Hilbert space (RKHS) regression and support vector regression (SVR). These methods rely on kernel functions for building a covariance structure among target observations based on the similarity (or dissimilarity) of their predictor variables (Gianola *et al*. 2006; Gianola and van Kaam 2008; de los Campos *et al*. 2010). This approach is particularly appealing because the “kernel trick” allows computations to be performed on an *N*-dimensional space, generally offering higher computational efficiency when *N* (number of observations) is smaller than *p* (number of markers).

Methods such as BLUP and GBLUP can be viewed as special cases of RKHS regression (de los Campos *et al*. 2009; Morota and Gianola 2014). This latter method has been expanded to more complex situations arising in animal and plant breeding (Momen *et al*. 2021; Montesinos-López *et al*. 2022). SVR is also a powerful alternative for genomic prediction of complex traits in both plant and animal organisms (Long *et al*. 2011; Azodi *et al*. 2019). Nonetheless, its connection with other kernel-based methods (e.g. GBLUP) is not straightforward, given the use of hinge loss functions and constrained optimization problems. Hence, generalizations of SVR to broader scenarios in the genomic prediction context are less common.

The objective of this study was to propose and investigate the performance of nonlinear kernels for MT-GP using SVR models. The high computational burden for hyperparameter fine-tuning is alleviated by considering trait-common values for the model bias, regularization parameter, and epsilon constant, leading to an optimization problem that can be handled with standard quadratic programming. The *H*-dimensional hyperparameter space (with *H* = 2 + *t*^2^, where *t* is the number of traits) is then optimized via a stochastic evolutionary algorithm. A case study with carcass traits in commercial broilers is presented.

## Materials and methods

### (Quasi) multitask support vector regression

Let $\;D=\{(y_{i},\;m_{i}^{T}),\;i\;=\;1,\;2,\ldots ,N\}$ be a training data set, with $y_{i}\varepsilon \{\;\text{–}\infty ,+\infty \}$ representing a real-valued observation for the *i*th individual and $m_{i}^{T}$ a *p*-dimensional row vector of single nucleotide polymorphisms (SNPs), coded as 0, 1, and 2, for genotypes AA, AB, and BB, respectively. This data set can be represented in matrix form as $M_{\left(Nxp\right)}$ and $y_{\left(Nx1\right)}$. Following this notation, the general SVR in its primal form can be written as:


(1) $$y=b_{0}+\;\phi (M)\beta +e,$$

in which β is the vector of unknown regression weights, ϕ(M) represents a linear or nonlinear mapping of the genotypes’ matrix in the feature space, $b_{0}$ is the model bias, and **e** is the vector of random errors.

A common cost function for SVR optimization is the ε-insensitive, a hinge loss function that penalizes absolute errors larger than a given constant “ε.” The optimal solutions for equation (1) are found by minimizing the following regularized loss function (Vapnik 1995):


(2) $$\;\frac{1}{2}\beta^{T}\beta +\;C[L_{\epsilon }(e^{T})]1_{n},$$

where $L_{\epsilon }=\{\begin{array}{c} 0,\;if\;|e_{i}|\;\leq \varepsilon \\ |e_{i}|-\;\varepsilon ,\;\mathrm{otherwise}\; \end{array}$, $e_{i}=\;y_{i}-\phi (m_{i}^{T})\beta -b_{0}$, *C* is a positive regularization hyperparameter that controls the penalty imposed on residuals larger than the epsilon margin (*ε*), and $1_{n}$ is an *N* × 1 vector of ones. The $L_{\epsilon }$ loss function is more conveniently expressed by introducing slack variables ($\xi_{i}$ and $\xi_{i}^{*}$), with $\xi_{i}$ > 0 and $\xi_{i}^{*}$ = 0 for positive errors and $\xi_{i}$ = 0 and $\xi_{i}^{*}$ > 0, otherwise. Plugging $\xi_{i}$ and $\xi_{i}^{*}$ into equation (2) yields the following constrained optimization problem:


(3) $$\arg\;\min\;\frac{1}{2}\beta^{T}\beta +\;C(\xi \;+\;\xi^{*}{)}^{T}1_{n},$$

$$s.t\;\{\begin{array}{c} y-\phi (M)\beta -b_{0}\;\leq \;\varepsilon_{n}+\;\xi \;\\ \phi (M)\beta +b_{0}-\;y\;\leq \;\varepsilon_{n}+\;\xi^{*}\;\\ \xi_{i},\;\xi_{i}^{*}\;\geq 0,\;\mathrm{for}\;i\;=\;1,2,\ldots ,N\; \end{array},$$

where ξ and $\xi^{*}$ are vectors of slack variables of dimension *N* × 1 and $\varepsilon_{n}=\varepsilon 1_{n}$ (i.e. the product between the epsilon constant and an *N* × 1 vector of ones).

It can be shown that minimizing the primal objective function in equation (3) is equivalent to maximizing its Lagrangian form (Appendix A):


(4) $$-\frac{1}{2}(\alpha -\alpha^{*}{)}^{T}K(\alpha -\alpha^{*})-\;(\alpha +\alpha^{*}{)}^{T}\;\varepsilon_{n}+(\alpha -\alpha^{*}{)}^{T}y,$$

$$s.t\;\{\begin{array}{c} 0\leq \;\alpha_{i},\;\alpha_{i}^{*}\;\leq C,\;i\;=\;1,2,\ldots ,N\;\;\\ {\left(\alpha -\alpha^{*}\right)}^{T}1_{n}=0 \end{array},$$

in which α and $\alpha^{*}$ are *N* × 1 vectors of positive Lagrange multipliers and $K=k\phi (M)\phi (M^{T}{)}_{H}$ is a (semi)positive definite kernel matrix in the Hilbert space. One common kernel choice is the radial basis function (RBF), with elements computed as $\;k_{ij}=\exp\;(-{\left\|m_{i}^{T}-m_{j}^{T}\right\|}^{2})$, where θ is the kernel bandwidth parameter and ∥.∥ denotes the Euclidean norm.

This model can be extended to a MTL problem by applying a generalized sequential minimal optimization (SMO) algorithm which solves *t* inequality constraints at once (Cai and Cherkassky 2012). Although this is a flexible approach and with a potentially higher generalization ability than the single-task SVR, the number of hyperparameters in such an approach largely scales with the number of tasks (traits), which can turn the model tuning process prohibitive for high-dimensional problems (e.g. genomic prediction). To take advantage of the joint information between correlated traits in a computationally efficient manner, the following data expansion scheme is proposed:


(3) $$\begin{array}{rl} \arg\;\mathrm{ma}x_{\alpha ,\alpha^{*}} & -\frac{1}{2}{\left[\begin{array}{c} \alpha^{\left(1\right)}-\alpha^{*(1)}\\ \alpha^{\left(2\right)}-\alpha^{*(2)}\\ \begin{array}{c} \vdots \\ \alpha^{\left(t\right)}-\alpha^{*(t)} \end{array} \end{array}\right]}^{T}Q\left[\begin{array}{c} \alpha^{\left(1\right)}-\alpha^{*(1)}\\ \alpha^{\left(2\right)}-\alpha^{*(2)}\\ \begin{array}{c} \vdots \\ \alpha^{\left(t\right)}-\alpha^{*(t)} \end{array} \end{array}\right]-\;{\left[\begin{array}{c} \alpha^{\left(1\right)}+\alpha^{*(1)}\\ \alpha^{\left(2\right)}+\alpha^{*(2)}\\ \begin{array}{c} \vdots \\ \alpha^{\left(t\right)}+\alpha^{*(t)} \end{array} \end{array}\right]}^{T}\\ & \varepsilon_{n}⊗1_{t}+{\left[\begin{array}{c} \alpha^{\left(1\right)}-\alpha^{*(1)}\\ \alpha^{\left(2\right)}-\alpha^{*(2)}\\ \begin{array}{c} \vdots \\ \alpha^{\left(t\right)}-\alpha^{*(t)} \end{array} \end{array}\right]}^{T}\left[\begin{array}{c} y^{*\left(1\right)}\\ y^{*\left(2\right)}\\ \begin{array}{c} \vdots \\ y^{*\left(t\right)} \end{array} \end{array}\right]\\ & s.t\;\{\begin{array}{c} 0\leq \;\alpha_{i}^{\left(k\right)},\;\alpha_{i}^{*(k)}\leq C,\;i\;=\;1,2,\ldots ,N\;\;\\ {\left[\alpha^{\left(k\right)}-\alpha^{*(k)}\right]}^{T}1_{n}=0,\;k\;=\;1,\;2,\;\ldots \;t\; \end{array}, \end{array}\;$$

where *t* refers to the number of traits, $\alpha^{\left(k\right)}$ and $\alpha^{*(k)}$ are the vectors of positive Lagrange multipliers for trait *k*, $1_{t}$ is a *t*-dimensional vector of ones, ⊗ is the Kronecker product, $[y^{*(1)},y^{*(2)},\ldots ,y^{*(t)}{]}^{T}$ are vectors of observed values for *t* traits standardized to the same scale, and **Q** is a block symmetric matrix, partitioned into *t*^2^ blocks of dimension *N* × *N*, each. We propose a MT scaled RBF kernel, as follows:


(4) $$Q=\left[\begin{array}{ccc} \exp\;(-\theta_{11}D\#;D\;p^{-1}) & \cdots & \rho_{1t}\;\exp\;(-\theta_{1t}D\#;Dp^{-1})\\ \vdots & \ddots & \vdots \\ \rho_{t1}\exp\;(-\theta_{t1}D\#;Dp^{-1}) & \cdots & \exp\;(-\theta_{tt}D\#;Dp^{-1}) \end{array}\right],$$

where **D** is an *N* × N Euclidean distance matrix (EDM) for the markers’ vectors, considering all genotyped individuals for at least 1 of the traits analyzed, #; is the Hadamard product, *p* is some scalar representing the number of predictor variables (e.g. number of SNPs), $\theta_{kk}$ and $\theta_{kk^{\prime }}$ are trait-specific and trait-common bandwidth hyperparameters, and $\rho_{kk^{\prime }}$ is some constant between −1 and 1 indicating the magnitude and direction of the association between traits *k* and $k^{\prime }$.

Using the dual formulation in equation (3), the function for predicting yet-to-be-seen observations depends solely on the mapping kernel and can be written as:


(5) $$f(x_{i})=\;\overset{(N\;x\;t)\;}{\underset{\;j=1}{\sum }}\;(\alpha_{j}^{\left(k\right)}-\;\alpha_{j}^{*(k)})\{Q_{ij}\}+b_{0},\text{with}\;k=1,2,\ldots t,$$

in which $Q_{ij}$ maps the relationship in the feature space between individual *i* and every column *j* in the matrix **Q**. In equation (5), typically only a subset of data points will have nonnull Lagrange multipliers, the so-called support vectors (SVs). The predicted value $f(x_{i})$ is then returned to the original scale. Notice that the bias ($b_{0}$) value, the regularization hyperparameter (*C*), and the epsilon threshold (ε) are assumed to be constant for all traits in equations (3) and (5); consequently, one can fit this extended SVR model with the standard SMO algorithm, available in most kernel-based method libraries, just by precomputing the block matrix Q. Given the simplifications, this model can be considered as a (quasi) multitask support vector regression (QMTSVR) as it can be used for expanding the observations for a target response (the trait of interest) by aggregating the complementary information from *t* − 1 correlated traits. This model has $2+\;t^{2}$ hyperparameters to be tuned, the global *C* and ε constants besides the kernel bandwidth parameters $\left(\theta_{kk^{\prime }}\right)$, and the association constants ($\rho_{kk}$) in Q. If one fixes $\rho_{kk^{\prime }}$ based on a priori information (e.g. the phenotypic or genetic correlation coefficient between traits *k* and $\;k^{\prime }$), the hyperparameter space reduces to *t*[*t* + 1]/2.

### Weighted QMTSVR

The block matrix Q in equation (4) can be further expanded to account for the loci-specific effects in each trait combination by replacing **D** with a weighted Euclidean distance matrix (**WD**), with elements computed as:


(6) $$\;wd_{ij}^{\left(kk^{\prime }\right)}=\sqrt{\overset{p}{\underset{l=1}{\sum }}\;w_{l}^{\left(kk^{\prime }\right)}{\left(m_{il}-m_{\;jl}\right)}^{2}}$$

where $wd_{ij}^{\left(kk^{\prime }\right)}$ is the weighted Euclidean distance between individuals *i* and *j* in the *N* × *N* block, computed for traits *k* and $k^{\prime }$ (with $k=k^{\prime }$ or $k\neq k^{\prime }$ and *k* = 1, 2, … *t*), $m_{il}$ and $m_{\;jl}$ are the marker genotypes for individuals *i* and *j* at *locus l*, *p* is the total number of *loci*, and $w_{l}^{\left(kk^{\prime }\right)}$ is the weighting factor for *locus l* considering the traits *k* and $k^{\prime }$. In this approach, *t*[*t* + 1]/2 different **WD** must be computed for plugging into **Q** and the constant *p*^−1^ is replaced with ${\left(\overset{p}{\underset{l=1}{\sum }}\;w_{l}^{\left(kk^{\prime }\right)}\right)}^{-1}$.

We computed the weighting factor for locus *l* as $w_{l}^{\left(kk^{\prime }\right)}=\;\frac{\left|2(1-\;q_{l})q_{l}\delta_{lk}\delta_{lk^{\prime }}\right|}{\max[\left|2(1-\;q_{l})q_{l}\delta_{lk}\delta_{lk^{\prime }}\right|]}$, where $q_{l}$ is the frequency of the second allele in locus *l*, $\delta_{lk}$ and $\delta_{lk^{\prime }}$ are effects of the *l*th locus on traits *k* and $k^{\prime }$, and $\max[\left|2(1-\;q_{l})q_{l}\delta_{lk}\delta_{lk^{\prime }}\right|]$ returns the maximum variance value found across all loci when $k=k^{\prime }$ or maximum absolute covariance value when $k\neq k^{\prime }$. Using this approach, $w_{l}^{\left(kk^{\prime }\right)}$ ranges between 0 and 1 for SNPs with low and high relative (co)variances, respectively. The coefficients $\delta_{lk}$ and $\delta_{lk^{\prime }}$ were obtained SNP-wise by solving a multivariate multiple regression. To account for potential population structure, the first 30 principal components of the genomic relationship matrix (**G**; VanRaden 2008) were also used as explanatory variables.

### Benchmark methods

We compared the predictive ability of the proposed approach with conventional single-trait (ST) and MT genomic prediction models; these methods are briefly described below.

#### GBLUP

The ST GBLUP can be described as follows:


(7) $$y=\;1_{n}\mu +Zu+e,$$

where y is a *N* × 1 vector of observations, $1_{n}$ is a *N* × 1 vector of ones, μ is the model intercept, **Z** is a design matrix connecting observations to the genomic breeding values (DGVs), u is the vector of predicted DGVs, and e is a *N* × 1 vector of residual terms.

Under a Bayesian paradigm, the following priors are assumed for the model unknowns: p(μ) ∼ constant, *p*(**u**|**G**, $\sigma_{u}^{2}$) ∼ *N*(**0**, **G**$\sigma_{u}^{2}$), *p*(**e**|$\sigma_{e}^{2}$) ∼ *N*(**0**, **I**$\sigma_{e}^{2}$), and *p*($\sigma_{i}^{2}$) ∼ $\mathrm{inv}\;\chi^{2}$($\sigma_{i}^{2}|S_{i},\;df_{i}$), where **G** is the genomic relationship matrix (VanRaden 2008), $\sigma_{u}^{2}$ is the genomic additive variance, **I** is an *N* × *N* identity matrix, $\sigma_{e}^{2}$ is the residual variance, and $S_{i}\;$ and $df_{i}$ are, respectively, the scale and degrees of freedom hyperparameters assumed for $\sigma_{i}^{2}$ (*i* = *u* or *e*).

#### MTGBLUP

The MT counterpart for the GBLUP has the following form:


(8) $$Y=\;1_{n}⊗\mu_{t}+Z_{t}U+E,$$

where **Y** is a matrix of response variables of dimension *N × t*, $\mu_{t}$ is a row vector of trait-specific intercepts, $Z_{t}$ is a design matrix for the random additive effects, U is an *N* × *t* matrix of predicted DGVs, and E is a matrix of residuals (individuals in rows and traits in columns).

The prior distributions for the model unknowns are assumed independent and can be represented as follows (Perez-Rodrıguez and de los Campos 2022):

*p*($\mu_{t}^{T})$ ∼ MVN(**0**, $I_{t}m$), *p*($u_{*}|\Sigma_{u},\;G$) ∼ MVN(**0**, $\Sigma_{u}⊗\;G$), *p*($e_{*}|\Sigma_{e}$) ∼ MVN(**0**, $\Sigma_{e}⊗\;I$), and *p*($\Sigma_{i}$) ∼ IW($\Sigma_{i}|S_{i},\;df_{i}$), where MVN(**0**, $I_{t}m$) represents a diffuse prior, with *m* equal to 1,000 times the maximum variance of each phenotype; $u_{*}$ and $e_{*}$ are, respectively, vectors with the columns of U and **E** stacked; $\Sigma_{u}$ and $\Sigma_{e}$ are *t* × *t* matrices of genetic and residual (co)variances, respectively; $S_{i}$ is the prior scale matrix; and $df_{i}$ is the prior degrees of freedom (*i* = *u* and *e*).

#### ST and MT RKHS regression (RKHS and MTRKHS)

These methods have the same general formulation previously presented for the GBLUP and MTGBLUP models. The key difference between the GBLUP and RKHS regression (and their MT counterparts) is the replacement of the **G** matrix with the same nonlinear RBF kernel (**K**) used to fit the SVR method. Accordingly, prior distributions of animal random effects are given by *p*(**u**) ∼ *N*(0, **K**$\sigma_{u}^{2}$) and *p*($u_{*}$) ∼ MVN(0, $\Sigma_{u}⊗\;K$) for the univariate and multivariate cases, respectively.

#### ST BayesC

The general form of a Bayesian regression model is represented as follows:


(9) $$y=\;1_{n}\mu +M\alpha +e,$$

where M is the *N × p* matrix of marker genotypes and α is a *p ×* 1 vector of allele substitution effects. The BayesC (BC) assumes a spike–slab prior for all marker effects, which refers to a mixture distribution comprising a point of mass at zero, with probability *π*, and a univariate normal distribution with probability 1 − *π* (Habier *et al*. 2011).

The joint density of the mixture prior is given by *p*($\alpha ,\sigma_{\alpha }^{2},\;\pi )=\left\{\overset{p}{\underset{\;j=1}{\prod }}\;[\pi (\alpha_{j}=0)+(1-\pi )N(0,\sigma_{\alpha }^{2})]\times \mathrm{inv}\;\chi^{2}(\sigma_{\alpha }^{2}|S_{\alpha },\;df_{\alpha })\times \mathrm{Beta}(\pi |p_{o},\pi_{0})\right\}$, where $\sigma_{\alpha }^{2}$ is a common variance for the allelic effects, $S_{\alpha }$ and $df_{\alpha }$ are the prior values for the scale and degrees of freedom parameters, $p_{o}>0\;$ and $\pi_{0}[0,1]$ are the prior shape parameters of the beta distribution, and *p*(μ) and *p*$\left(\sigma_{e}^{2}\right)$ are defined as before in equation (7).

#### MTBC

A multivariate Bayesian regression model can be represented in matrix form as follows:


(10) $$Y=1_{n}⊗\mu_{t}+MB+E$$

where **B** is a *p* × *t* matrix of allele substitution effects (trait-specific solutions in rows and traits in columns) and the remaining terms are defined as in equation (8). One possible multivariate expansion for the BC is to define a mixture prior with a point of mass at zero and a multivariate normal slab. The entries of *B* are represented as $A^{*}\#;D^{*}=\;\alpha_{\;jk}^{*}\times d_{\;jk}^{*}$, the product of a normal random variable ($\alpha_{\;jk}^{*}$) and a dummy variable ($d_{\;jk}$) which controls whether the *j*th marker has a nonnull effect on the *k*th trait. Thus, the prior distribution for the marker effects can be represented as $p(A^{*},D^{*})=\left\{\overset{p}{\underset{\;j=1}{\prod }}\;\mathrm{MVN}(\alpha_{\;jk}^{*}|0,\Sigma_{\alpha^{*}})\right\}\times \left\{\overset{p}{\underset{\;j=1}{\prod }}\;\overset{t}{\underset{k=1}{\prod }}\;\pi_{k}^{d_{\;jk}^{*}}{\left(1-\pi_{k}\right)}^{1-d_{\;jk}^{*}}\right\}$, where $\Sigma_{\alpha^{*}}$ is the *t × t* (co)variance matrix for the markers’ effects, with $\Sigma_{\alpha^{*}}∼\;\mathrm{IW}(\Sigma_{\alpha^{*}}|S_{\alpha^{*}},\;df_{\alpha^{*}})$. The parameters {$\pi_{1},\;\pi_{2},\;\ldots ,\;\pi_{t}$} represent the proportion of nonnull effects in each trait, with $p(\pi_{1},\;\pi_{2},\;\ldots ,\;\pi_{t})\propto \overset{t}{\underset{k=1}{\prod }}\;\pi_{k}^{a_{1}}(1-\pi_{k}{)}^{a_{2}}$, where $a_{1}$ and $a_{2}$ are the prior shape parameters of the beta distribution (Perez-Rodrıguez and de los Campos 2022).

#### Linear SVR

Lastly, we also assessed the prediction performance of the ST SVR with a linear kernel [linear SVR (SVR_Lin)]. The optimization of this method is performed as represented in equation (4). The only difference is that SVR_Lin uses the genomic relationship matrix (**G**) as the kernel instead of the RBF kernel (**K**).

### Phenotypic and genotypic data

The data set used in this study was provided by Cobb-Vantress Inc. (Siloam Springs, AR) and comprised phenotypic observations from broiler purebred lines, bred in 23 overlapping mating generations (MG, MG1–MG23). The phenotypic data included 2 carcass traits (*CT1* and *CT2*) and 2 in vivo traits [*Growth* and feed efficiency trait (*FE*)]. Before genomic analyses, pedigree-based breeding values (EBV) were estimated for all traits using all available observations (*N* = 92,952 for *Growth*, 48,816 for *FE*, and 11,002 for both *CT1* and *CT2*). The EBV for *CT1*, *CT2*, and *FE* were estimated with a 2-trait animal model, including *Growth* as a regressor trait for attenuating selection bias. The pedigree-based analysis considered the effects of contemporary group (GC) and sex (only for *Growth* and *FE*) as fixed. The CG combined the effects of the hatch, source, and MG. The (co)variance components of the pedigree-based analyses were estimated using the AIREML algorithm, available in the BLUPF90 suite programs (Misztal *et al*. 2018).

For the genomic analyses, the EBVs were deregressed using the method proposed by Garrick *et al*. (2009). This data processing step was performed to avoid double-counting of the parent average contribution in the training and testing sets. The deregressed proofs (DRPs) were used as pseudophenotypes in the genomic prediction analyses. Only animals with DRP reliability higher than 0.4 for all traits were retained. This exclusion criterion was adopted to reduce heteroskedasticity due to unequal prediction error variances for the expected breeding values. The number of genotyped individuals that attained such criteria was 6,011. The genotypic data were obtained using a moderate-density Illumina (San Diego, CA) chip, containing 60k SNP markers. For the genotypes’ quality control (QC), only SNPs with minor allele frequency >0.05 and call rate > 95% were retained. All data editing procedures and genotypic QC were performed in the R environment (R Core Team 2021).

#### Model validation and comparison criteria

The predictive ability of the ST and MT models was assessed by splitting the data into training and testing data sets according to the chronological information of MG (i.e. forward-in-time validation). The animals from MG1 to MG19 (*N* = 4,186) were used for fine-tuning and training the models, whereas the data from MG20 to MG23 (*N* = 1,825) were held out as the testing set. In the ST prediction, only observations for the target trait available in the training set were used for fitting the models. For the MT analyses, each target trait (*CT1* and *CT2*) was analyzed with an indicator trait using a 2-trait model (i.e. *CT1* × *Growth* and *CT2* × *FE*).

As discussed in Burgueño *et al*. (2012), we assessed the predictive ability of MT models using 2 validation designs (CV1 and CV2). In CV1, individuals in the testing data (MG20–MG23) did not have observations available for any trait, while in CV2, the information for the indicator trait was available in both training and testing sets. CV1 mimics a scenario where the interest is to predict the performance of young animals with available genotypes, but which have not been measured for any trait yet. Conversely, CV2 represents a scenario where individuals have been already tested for secondary traits that are early expressed and/or are easier to measure. According to the method (univariate or multivariate) and validation design (CV1 and CV2), the assessed genomic prediction models can be summarized as GBLUP, BC, RKHS, SVR, SVR-Lin, WSVR, MTGBLUP-CV1, MTBC-CV1, MTRKHS-CV1, QMTSVR-CV1, weighted QMTSVR (WQMTSVR)-CV1, MTGBLUP-CV2, MTBC-CV2, MTRKHS-CV2, QMTSVR-CV2, and WQMTSVR-CV2.

The first criterion used to assess the models’ predictive ability was the corrected accuracy (ACC), defined as $\;r(y_{1_{\mathrm{tst}}},{\hat{u}}_{1_{\mathrm{tst}}})/\sqrt{re_{y_{1}}^{2}}$, where r(.,.) represents the Pearson correlation coefficient, $y_{1_{\mathrm{tst}}}\;$ is the vector of observed DRPs for the target trait (*CT1* or *CT2*) in the testing set, ${\hat{u}}_{1_{\mathrm{tst}}}$ is the vector of predicted DRPs for the target in the testing set, and $re_{y_{1}}^{2}$ is the average DRP's reliability for the target trait. The predictive ability was also assessed in terms of relative root-mean-square error ($\mathrm{RMS}E^{*}$), computed as $\;\mathrm{RMS}E^{*}=\mathrm{RMSE}/\sqrt{\sigma_{y_{1_{\mathrm{tst}}}}^{2}}$. In this expression, $\sigma_{y_{1_{\mathrm{tst}}}}^{2}$ is the sample variance of the target trait in the testing set, and the numerator is computed as $\mathrm{RMSE}=\;\;\;\sqrt{\frac{1}{N_{\mathrm{tst}}}{\left(y_{1_{\mathrm{tst}}}\;-\;{\hat{u}}_{1_{\mathrm{tst}}}\right)}^{T}(y_{1_{\mathrm{tst}}}-{\hat{u}}_{1_{\mathrm{tst}}})}$, where $N_{\mathrm{tst}}$ represents the number of observations in the testing set. As for the regular root-mean-squared error (RMSE), smaller values are preferable for standardized RMSE $\left(\;\mathrm{RMS}E^{*}\right)$. The prediction bias was quantified with the inflation coefficient (*b*), i.e. the slope of the linear regression of realized observations on predicted values in the testing set. Values of *b* above or below 1 represent upward- or downward-biased predictions, respectively.

Because both the focal ($y_{1}$) and the secondary ($y_{2}$) traits are measured on the same individuals, the predicted values in ${\hat{u}}_{1_{\mathrm{tst}}}$ can be influenced by environmental effects present in $y_{2_{\mathrm{tst}}}$ when fitting MT models using the CV2 validation design. This scenario can artificially inflate the estimated accuracy for MT methods in the CV2-style prediction, leading to suboptimal model selection (Runcie and Cheng 2019). To account for this potential bias, we also computed a parametric estimate of accuracy: $\mathrm{AC}C_{\mathrm{par}}=\;{\hat{h}}_{{\hat{u}}_{1_{\mathrm{tst}}}}\times {\hat{r}}_{g}(y_{1_{\mathrm{tst}}},{\hat{u}}_{1_{\mathrm{tst}}})$, in which ${\hat{h}}_{{\hat{u}}_{1_{\mathrm{tst}}}}$ is the square root of the heritability for the target trait predictions estimated in the testing set, and ${\hat{r}}_{g}(y_{1_{\mathrm{tst}}},{\hat{u}}_{1_{\mathrm{tst}}})$ is the estimated genetic correlation between $y_{1_{\mathrm{tst}}}$ and ${\hat{u}}_{1_{\mathrm{tst}}}$. The parameters ${\hat{h}}_{{\hat{u}}_{1_{\mathrm{tst}}}}$ and ${\hat{r}}_{g}(y_{1_{\mathrm{tst}}},{\hat{u}}_{1_{\mathrm{tst}}})$ were estimated via Bayesian inference by fitting a 2-trait mixed model (as described in equation (8)) to the testing set. This metric is based on the selection index theory and has been suggested as an alternative for removing the contribution of environmental covariance between ${\hat{u}}_{1_{\mathrm{tst}}}$ and $y_{1_{\mathrm{tst}}}$ from the calculation of accuracy (Runcie and Cheng 2019; Lopez-Cruz *et al*. 2020).

#### QMTSVR fine-tuning

We used a genetic algorithm (GA) to perform a heuristic search over the QMTSVR 6D parameter space (*C*, *ɛ*, $\;\theta_{11}$, $\;\theta_{12}$, $\theta_{22}$, and $\;\rho_{1t}$). This stochastic evolutionary algorithm compares a population of candidate models (with binary arrays representing different hyperparameter combinations or “chromosomes” in the GA terminology) according to their fitness scores (fs). The arrays representing the models with the best fs are then selected and crossed for composing the next generation. The resulting “child” arrays inherit features from parent chromosomes of the previous generation. The relevant parameters in the present GA implementation are the population size (PS), the number of generations (NG), crossover rate (CR), mutation rate (MR), and Tournament Size (tsize). The PS represents the number of models tested per generation; the CR controls the rate that a child’s chromosome will result from the crossing-over of 2 parents instead of being an identical copy of 1 of them. The MR is the probability of a single bit (or gene) on the binary array changing randomly, implying slight modifications to the current model. The selection operator in the GA was the tournament selection (TS), i.e. for each child chromosome to be created, tsize individuals are drawn at random from the current population and the one with the highest fs is selected for integrating the first spouse chromosome, and a second TS is then performed to select the other spouse chromosome. The GA parameters were fixed as NG = 30, PS = 25, CR = 0.9, MR = 0.05, and tsize = 4. The pseudocode for the current GA implementation is provided in Supplementary Fig. 1.

**Fig. 1.**
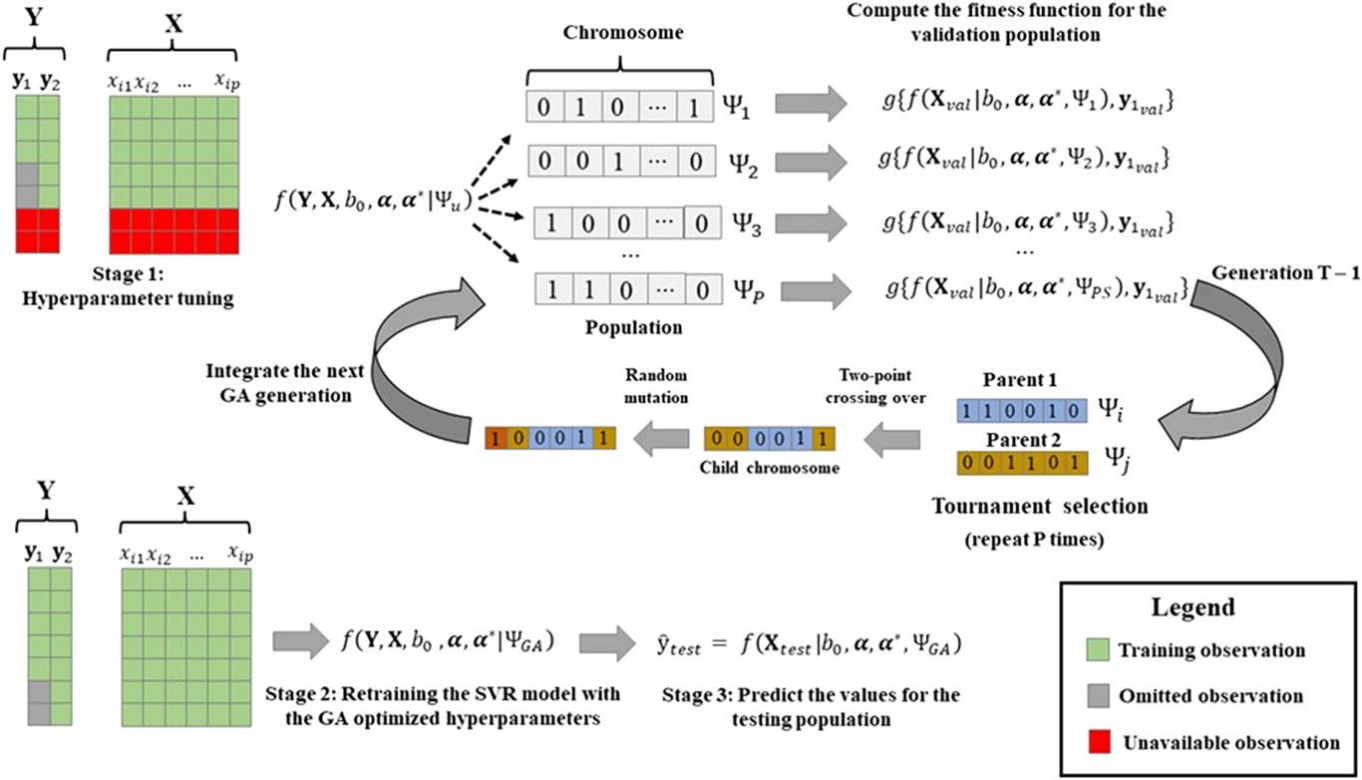
Optimization process of the QMTSVR method via a GA. During the GA-based fine-tuning, each hyperparameter set is coded as a binary array (the chromosome). The fitness function is computed for each chromosome based on the predictive ability that this hyperparameter set achieves in the validation sample (omitted during the GA-based optimization). The tournament selection operator selects a pair of individuals with the best fs of 2 subsets randomly drawn with replacements from the current population. A child chromosome is created for each chromosome pair sampled with the selection operator, and P new chromosomes are created for the next generation using the 2-point crossing-over and mutation processes. The *v* worst individuals (set of hyperparameters) of the current generation are replaced with the *v* best individuals from the previous generation. The GA algorithm repeats the process for *T* generations. The best hyperparameter set $\left(\Psi_{\mathrm{GA}}\right)$ is then used for retraining the model with all available observations.

The GA aimed to maximize the Pearson correlation between observed and predicted responses measured from the 17th to 19th MG in the training data. Therefore, during the hyperparameter fine-tuning, these observations (17th to 19th MG) were omitted within the GA optimization. After defining the best model hyperparameter configuration for each trait and training strategy, the SVR models were retrained with the full training data (MG1–MG19) for predicting the observations of the testing set (from MG20 to MG23). The hyperparameter search in the GA algorithm was restricted between the following intervals: 0.1 and 4 for *C*, 0.0001 and 0.1 for *ɛ*, 0.2 and 7 for the kernel bandwidths (*θ*_11_, *θ*_12_, and *θ*_22_), and between 0.1 and 0.3 for $\rho_{12}$. The GA-based fine-tuning and prediction of yet-to-be-seen observations with the QMTSVR method and CV2 design are illustrated in Fig. 1.

#### Software

The univariate and multivariate Bayesian shrinkage and variable selection models (GBLUP, MTGBLUP, RKHS, MTRKHS, BC, and MTBC) were fitted using the BGLR package (Perez and de los Campos 2014, 2022). For these methods, a total of 20,000 Gibbs sampling iterations were run, with a burn-in period of 5,000 and a sampling interval at every 5th iteration. The GA-optimized SVRs (SVR, WSVR, QMTSVR, and WQMTSVR) were fitted with the developing version of the *qmtsvr* package (Alves and Rosa 2022), which uses the *kernlab* library (Karatzoglou *et al*. 2004) as backend. SVR_Lin was trained directly in the *kernlab* library. To find the best hyperparameter combination for this method, a random grid search was performed in the training set. All analyses were run in an Intel Xeon CPU E5-2609 @2.40 GHz Linux workstation with 8 cores and 125-GB RAM.

## Results

### Summary statistics and genetic parameters

The mean ± standard deviation of the traits analyzed were 26.14 ± 2.46 (%), 1.64 ± 0.41 (%), 4660 ± 495.55 (g), and −16.84 ± 231.53 (g) for *CT1*, *CT2*, *Growth*, and *FE*, respectively. Heritability estimates (*h*^2^) obtained with the pedigree-based models were high for carcass traits, with values of 0.56 ± 0.01 for *CT1* and 0.63 ± 0.03 for *CT2*. For the indicator traits, heritability estimates were moderate, 0.34 ± 0.01 for *Growth* and 0.35 ± 0.01 for *FE*. The genetic and environmental correlations were, respectively, 0.23 and 0.47 between *CT1* and *Growth*, and 0.38 and 0.25 between *CT2* and *FE*.

**Fig. 2.**
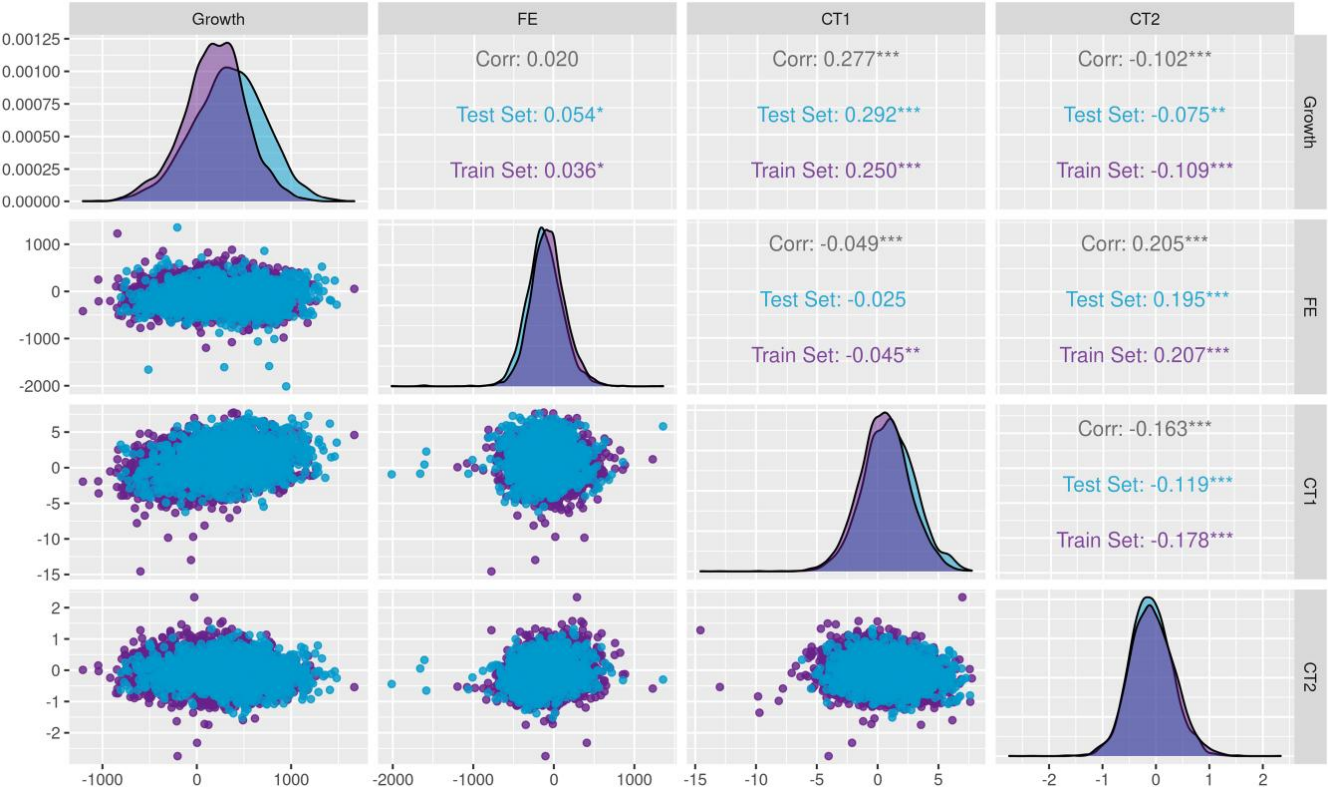
Scatter and density plots of deregressed expected breeding values for performance (*Growth* and *FE*) and carcass traits (*CT1* and *CT2*) measured in broiler chickens, according to training and testing data sets.

Figure 2 depicts that the deregressed EBVs (DRP) have bell-shaped normal distributions for all traits. The DRP averages were statistically different between training and testing sets for *Growth* (*P* < 0.05), evidencing that this trait is under selection, as suggested by the density plots (Fig. 2). The association between response variables (DRP) for the target and indicator traits was weak, as evidenced by the scatter plots (Fig. 2), with Pearson's correlation coefficients of 0.277 (*CT1* × *Growth*) and 0.205 (*CT2* × *FE*). Pearson's correlation among the remaining trait combinations ranged from null to weak (between −0.163 and 0.02^ns^). Additionally, there was no change in direction and magnitude for the correlation coefficients according to the training and testing samples.

### SVR hyperparameter optimization via GA

**Fig. 3.**
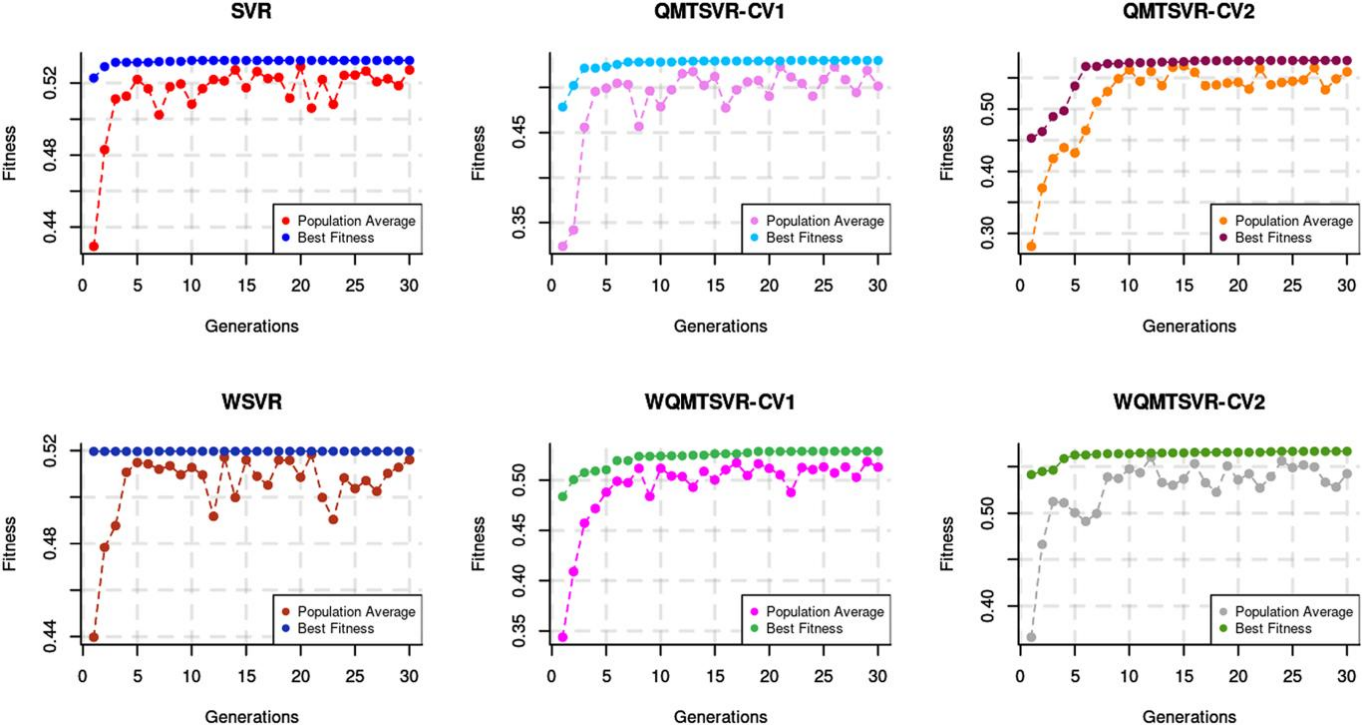
Best and average performance across generations of the GA optimization for SVR models with different hyperparameters. Models were trained using available information for carcass trait 1 (CT1) until the 16th MG. The vertical axis indicates the performance of selected models for predicting future CT1 observations from the 17th to 19th MG. QMTSVR stands for (quasi) multitask SVR, in which the information for *Growth* was also included for animals born until the 16th MG (QMTSVR-CV1) or considering that such information was available for all animals, including those from the testing sample (QMTSVR-CV2). WSVR, WQMTSVR-CV1, and WQMTSVR-CV2 are the corresponding models weighted with loci-specific information obtained in genome-wide association studies.

Figure 3 shows the trend for fs across GA generations for optimizing different SVR models trained for genome-enabled prediction of *CT1*. Note that the number of necessary generations to get rid of local minima increases as the evolutionary process proceeds. The highest optimization progress for *CT1* generally occurred at the early stages of the evolutionary process (up to the 10th generation of the GA), followed by a modest linear increase in the fs afterward (Fig. 3); a similar behavior was also observed for *CT2* (Supplementary Fig. 2). The random downward fluctuations for the population average fitness reflect unfavorable hyperparameter combinations caused by the crossover and mutation processes of the previous generation. These random perturbations are essential for exploring the search space in GA, leading eventually to models with favorable hyperparameter combinations that will pass through the next generations (Fig. 3).

The final hyperparameter values found with the GA varied markedly according to the trait, although values tended to converge to similar values within the same trait and method (Supplementary Table 1). In general, the trait-common bandwidth hyperparameter converged to large values (from 3.28 to 7.00), indicating that local kernels were generally preferred for integrating the genomic information between the 2 traits. Conversely, the kernel bandwidth for the block linking the target trait observations converged to relatively lower values (between 1.09 and 3.61), which indicates that the off-diagonal values in the block $Q_{11}$ have a larger influence on the target trait prediction.

**Fig. 4.**
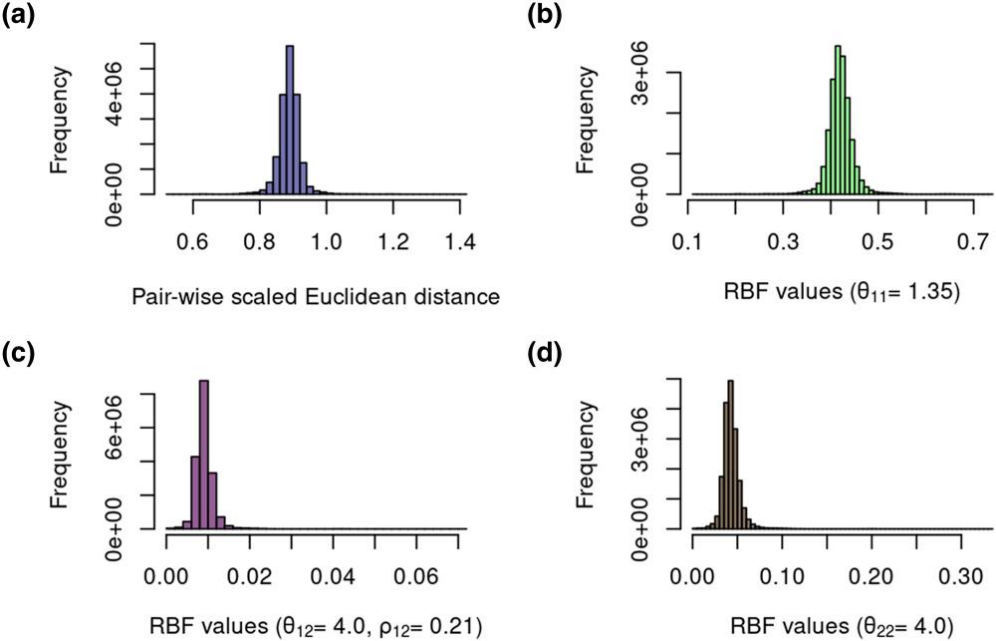
Histogram of off-diagonal elements of the scaled Euclidean distance matrix built for the genotyped individuals from a broiler line population a). Subplots b–d) illustrate the influence of different bandwidth hyperparameter values ($\theta_{11},\;\;\theta_{12},\;\;\mathrm{and}\;\theta_{22}\;$) and the weighting factor ($\rho_{12}$) on the off-diagonal elements of the multitask RBF kernel. It is shown that higher values for $\theta_{kk^{\prime }}$ decrease the overall similarity among individuals in the kernel.

This is better illustrated in Fig. 4, which depicts the influence of different kernel hyperparameters in the off-diagonal elements of the scaled Euclidean distance matrix. It can be noticed that the magnitude of the bandwidth hyperparameter ($\theta_{kk^{\prime }}$) is inversely proportional to the RBF kernel variance, with lower values meaning a higher influence of the data points toward each other in the feature space (Fig. 4b). Conversely, the similarity between 2 data points decreases as $\theta_{12}$ increases (Fig. 4c and d), and for the trait-specific kernel block, there is an additional shrinkage performed by the $\rho_{12}$ hyperparameter (Fig. 4c).

**Fig. 5.**
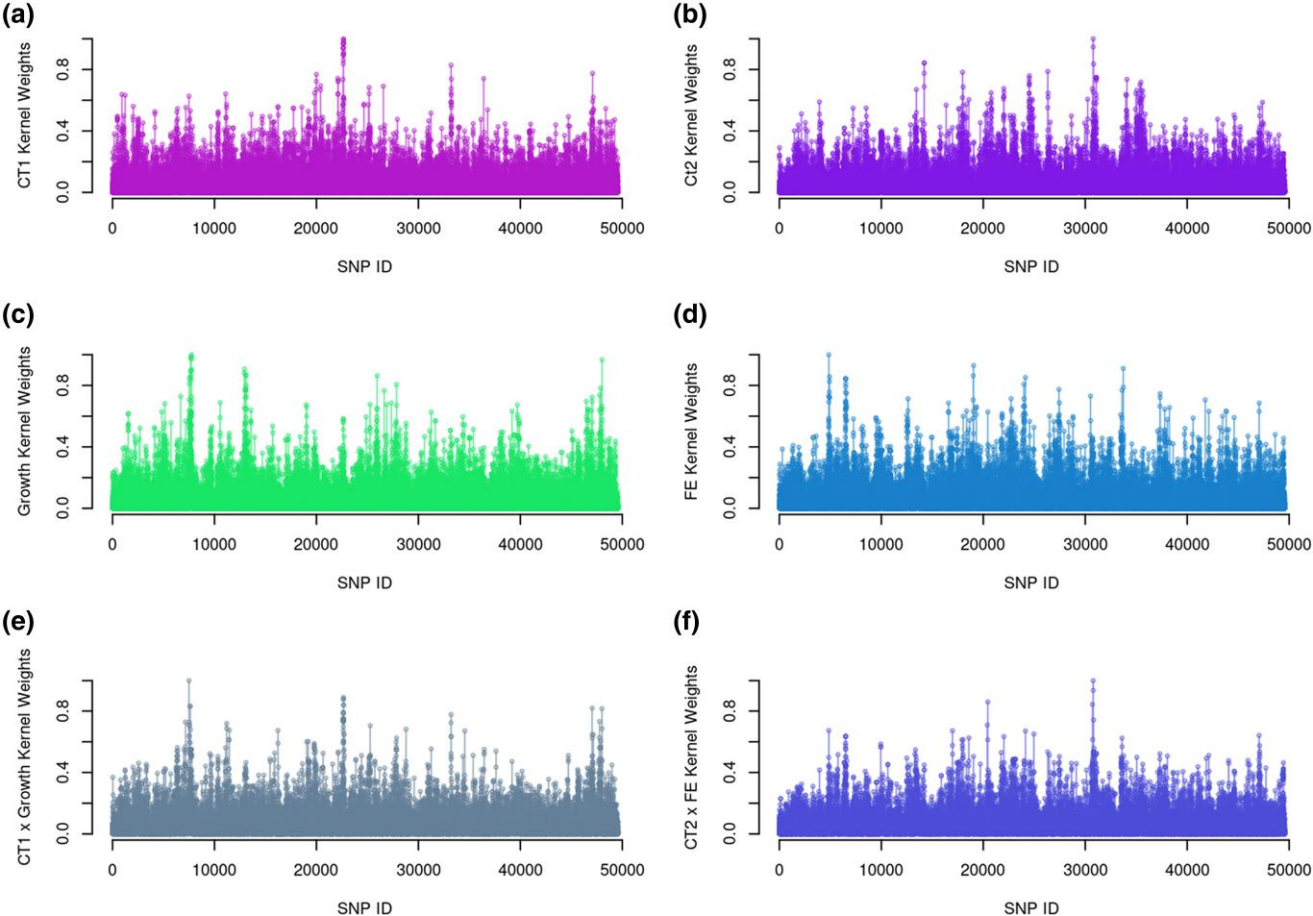
Weights for building trait-specific (a–d) and pleiotropic kernels (e and f) for the multi-task support vector regression methods. Weights used for the kernels were computed based on the relative SNP (co)variances, estimated with a multivariate multiple regression model.

Manhattan plots of the weighting factors used to build the **WD** matrices suggest a polygenic architecture of all traits analyzed, with many peaks observed across the genome (Fig. 5a–d). Overall, Fig. 5 suggests no evidence of pleiotropic quantitative trait *loci* with major effects in both target and indicator traits, which is somewhat expected, given the low genetic correlation coefficients estimated in the pedigree-based analyses.

### Predictive ability assessment

**Fig. 6.**
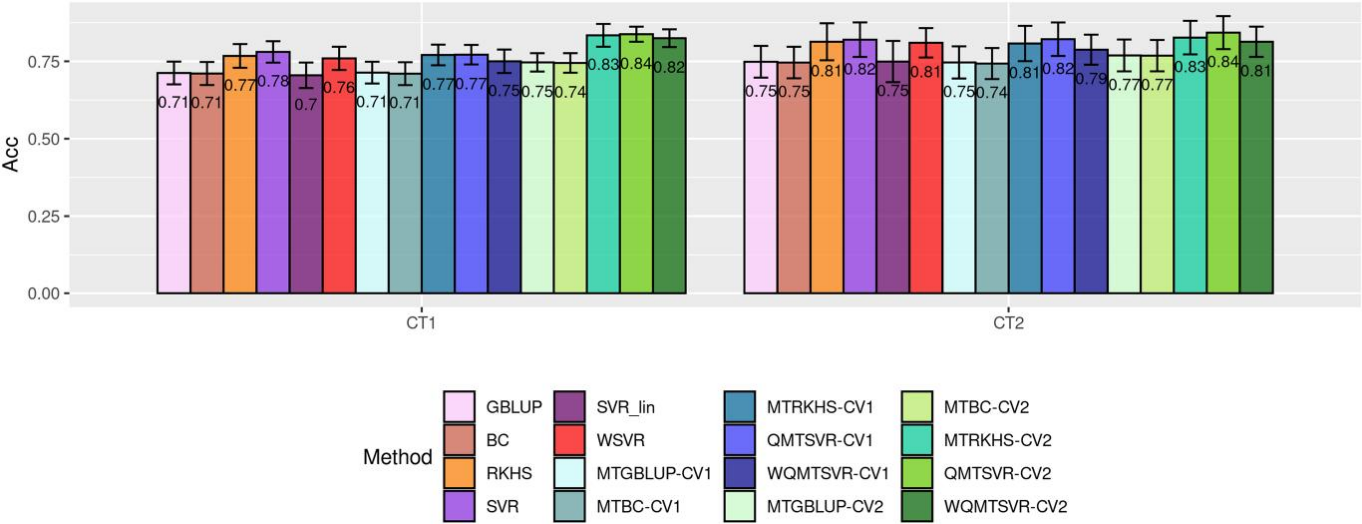
Predictive ACC of different ST and MT genomic prediction methods for deregressed expected breeding values of carcass traits (CT1 and CT2) measured in broiler chickens. Models were trained using available information for carcass traits until the 19th MG. The vertical axis indicates the accuracy in the testing set (from 20th to 23rd MG) according to the model and trait. MT models were trained using the available information on secondary traits in bitrait analyses (CT1 × Growth and CT2 × FE). CV1 indicates that the observations for the secondary trait were not available in the testing set. CV2 mimics a scenario where the secondary trait was measured in all animals (including those from the testing set). Error bars reflect the standard deviation of the accuracy across the 4 MG spanned by the testing set.

**Fig. 7.**
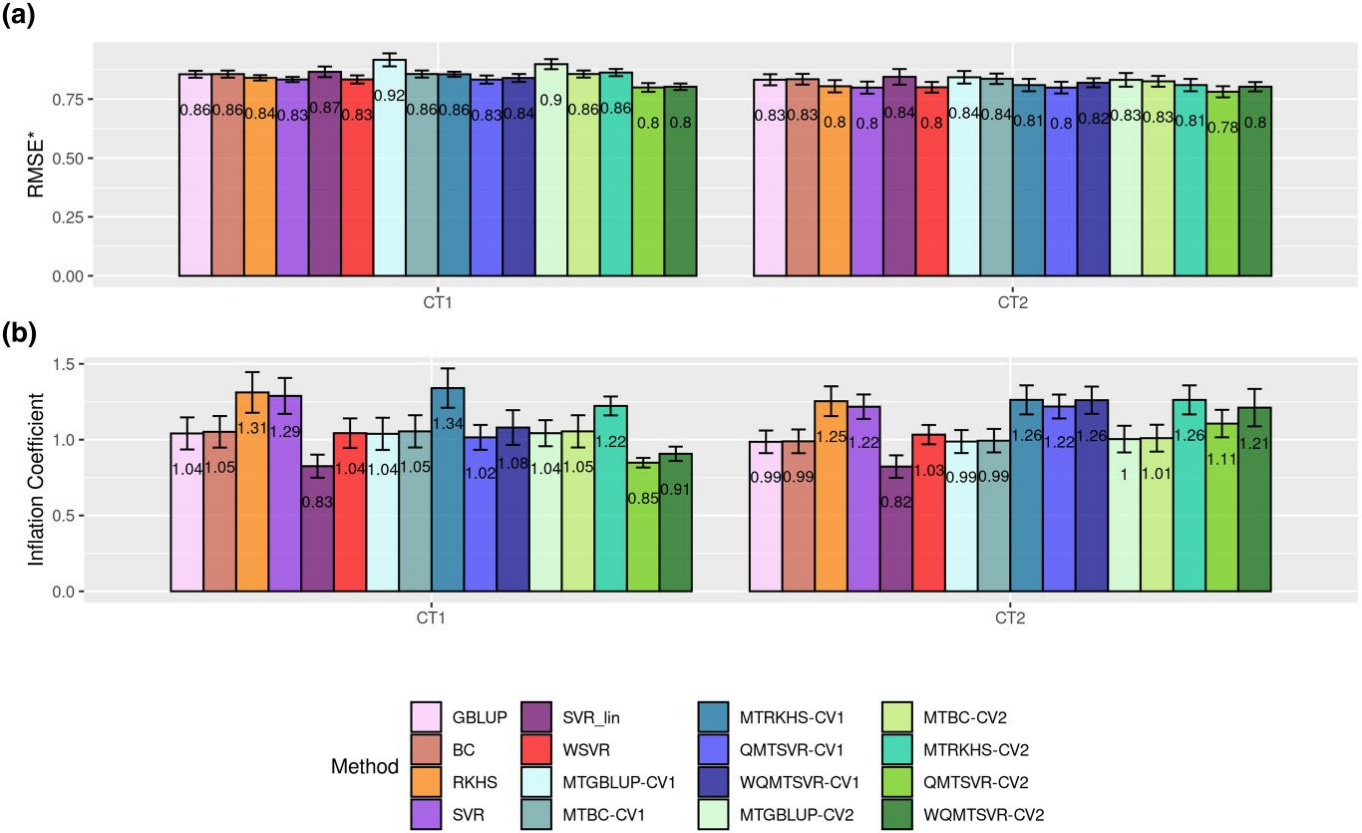
Performance comparison of single-trait (ST) and multiple-trait (MT) genomic prediction methods for de-regressed expected breeding values of two carcass traits (CT1 and CT2) in broiler chickens. Standardized root-mean-squared error (RMSE*; a) and inflation factor (b) are used as evaluation metrics. Models were trained using available information for carcass traits until the 19th mating generation (MG). The vertical axis indicates the predictive performance in the testing set (from 20th to 23rd MG) according to the model and trait. For MT models, the available information of secondary traits was analyzed jointly in bi-trait analyses (CT1 × Growth and CT2 × FE). CV1 indicates that the observations for the secondary trait were not available in the testing set. CV2 mimics a scenario where the secondary trait was measured in all animals (including those from the testing set). Error bars reflect the standard deviation of the predictive ability across the 4 MG spanned by the testing set.

The observed predictive ability metrics varied according to trait, model, and validation design (CV1 or CV2), ranging from 0.71 to 0.84 for ACC (Fig. 6), 0.80 to 0.92 for RMSE*, and between 0.83 and 1.34 for *b* (Fig. 7), considering *CT1*. Likewise, for *CT2*, the predictive ability metrics ranged from 0.75 to 0.84 for ACC (Fig. 6), from 0.78 to 0.84 for RMSE*, and from 0.82 to 1.26 for *b* (Fig. 7). ACC values were similar between *CT1* and *CT2*, which was expected since both traits have relatively high heritability, and the genomic analyses considered only the animals that surpassed a threshold of 0.4 for the DRP reliability in all traits. Results showed that the genomic prediction accuracy was close to the theoretical expectation from REML/BLUP.

When considering only the ST models, kernel methods incorporating nonlinear feature mapping (RKHS, SVR, and WSVR) had the highest ACC and the lowest RMSE, but worse *b* coefficient (in general, with higher deviation from 1) compared to models assuming linear effects only (GBLUP, BC, and SVR_Lin) (Figs. 6 and 7). There was an evident improvement in ACC by using the RBF kernel (**K**) relative to using the linear kernel (**G**) in the kernel-based regression methods (SVR vs SVR_Lin and GBLUP vs RKHS). On the other hand, predictive ability was rather similar between GBLUP and SVR_Lin, which indicates that using different loss functions (quadratic form in GBLUP vs hinge function in SVR) played a negligible impact on the predictive ability for both traits.

There was a slight improvement in ACC for all MT models compared to their respective univariate counterparts, although this difference was only observed when multivariate models were trained under the CV2 validation design (Fig. 6). The highest ACC (0.84) was achieved with QMTSVR-CV2, which delivered the same performance in both traits (Fig. 6). This corresponds to a relative gain of around 15 and 11% compared to GBLUP (and BC), 8 and 4% relative to RKHS (and MTRKHS-CV1), and 11 and 8% relative to MTGBLUP-CV2 (and MTBC-CV2), for *CT1* and *CT2*, respectively. No differences in ACC were found between QMTSVR-CV2 and MTRKHS-CV2 (Fig. 6). Nonetheless, considering the RMSE* and *b* metrics, there was a small advantage observed towards the QMTSVR-CV2 over the MTRKHS-CV2 (Fig. 7), with the second method presenting a higher relative prediction error and inflation coefficient farthest from 1 in both traits. Across multivariate methods, the MTGBLUP-CV1 method/validation delivered the worst RMSE* for *CT1*, while the smallest relative prediction error was achieved using QMTSVR-CV2 (Fig. 7a). In general, the relative prediction error was higher for *CT1* compared to *CT2*, which might be partially explained by the higher heritability estimated for *CT2*.

The ACC improvement from SVR to QMTSVR was only slight; however, the incorporation of the indicator trait information in the SVR was important for reducing prediction errors in both traits, as evidenced by the lower RMSE*. Furthermore, the QMTSVR also attenuated the bias observed for SVR predictions as reflected by the inflation factor closer to 1. On the other hand, incorporating information on *loci*-specific importance into the EDM computation did not improve the results achieved with the regular QMTSVR (Figs. 6 and 7).

**Fig. 8.**
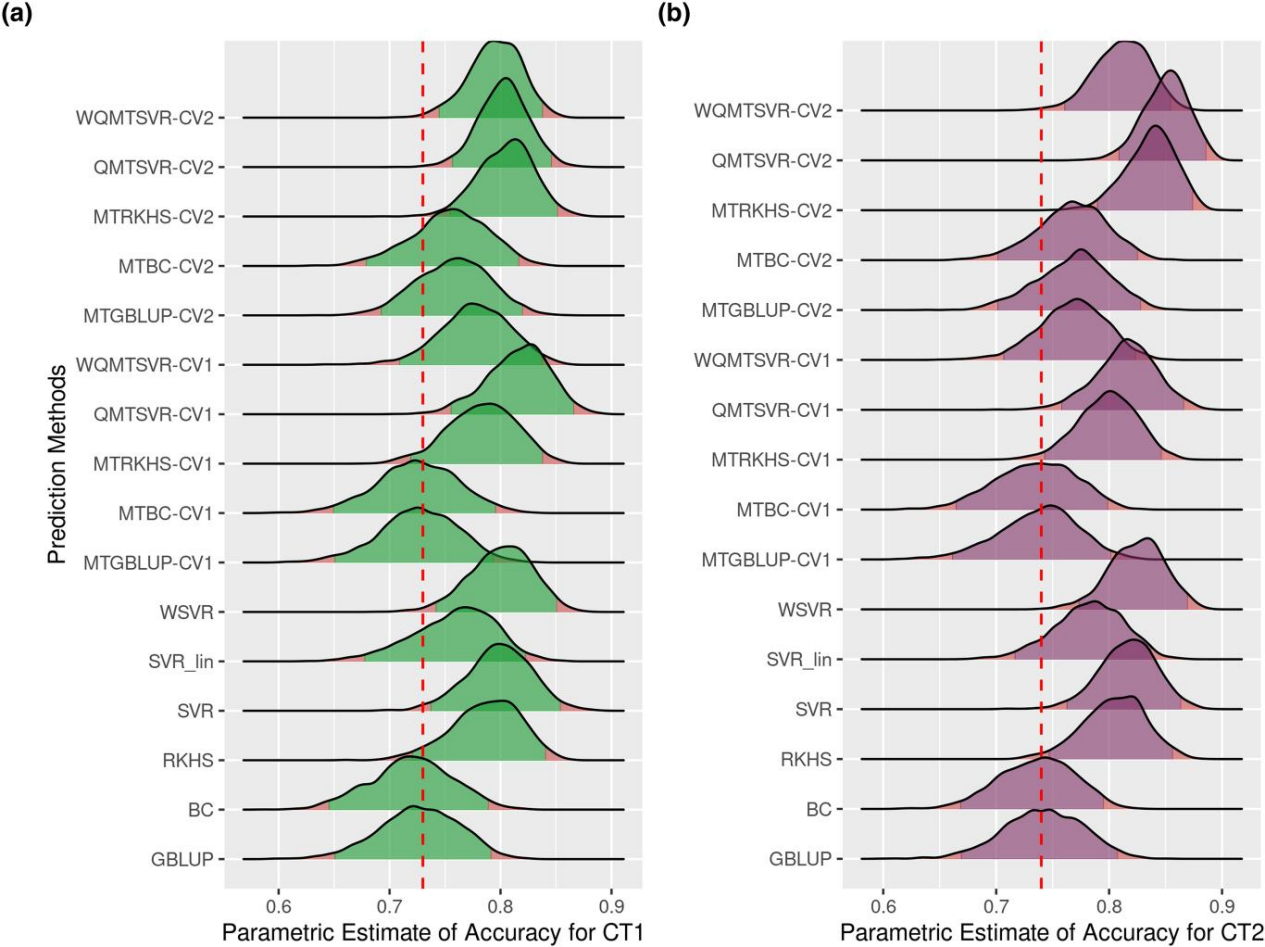
Posterior distributions of parametric estimates of accuracy for de-regressed expected breeding values of 2 carcass traits (a and b) in broiler chickens according to ST and MT genomic prediction methods. Models were trained using available information for carcass traits until the 19th MG and predictions were obtained for the testing set (from 20th to 23rd MG). For MT models, the available information of secondary traits was analyzed jointly in bi-trait analyses (CT1 × Growth and CT2 × FE). CV1 indicates that the observations for the secondary trait were not available in the testing set. CV2 mimics a scenario where the secondary trait was measured in all animals (including those from the testing set). The accuracy was estimated as the genetic correlation between predicted and observed values multiplied by the square root of heritability for predicted values. These parameters were estimated using only information in the testing set in a bi-trait mixed model analysis. Dashed lines indicate the posterior average of accuracy for GBLUP.

Since CV2-style predictions for the focal trait are prone to the environmental influence of indicator traits (Runcie and Cheng 2019), we also computed a parametric estimate of accuracy ($\mathrm{AC}C_{\mathrm{par}}$) for removing this possible bias. Figure 8 depicts the posterior distributions for $\mathrm{AC}C_{\mathrm{par}}$ according to the method and trait. Posterior averages of $\mathrm{AC}C_{\mathrm{par}}\;$ for *CT1* ranged between 0.72 ± 0.04 (BC) and 0.82 ± 0.03 (QMTSVR-CV1). Similarly, the $\mathrm{AC}C_{\mathrm{par}}\;$ averages ranged from 0.73 ± 0.04 (MTBC-CV1) to 0.85 ± 0.05 (QMTSVR-CV2) for *CT2*. Spearman correlations between ACC and the posterior average for $\mathrm{AC}C_{\mathrm{par}}$ were 0.86 and 0.95, for *CT1* and *CT2*, respectively. This indicates that despite these 2 metrics being highly associated, model selection may be sensitive to the chosen accuracy metric, especially for *CT1*. Credible intervals at 95% of probability (CI_95%_) for the $\mathrm{AC}C_{\mathrm{par}}\;$ of most MT methods (QMTSVR, WQMTSVR, and MTRKHS) did not include the reference value (average $\mathrm{AC}C_{\mathrm{par}}$ for GBLUP), especially under the CV2 validation design (Fig. 8). Therefore, the true $\mathrm{AC}C_{\mathrm{par}\;}$ value obtained by these MT models is unlikely to be equal to this reference point.

**Fig. 9.**
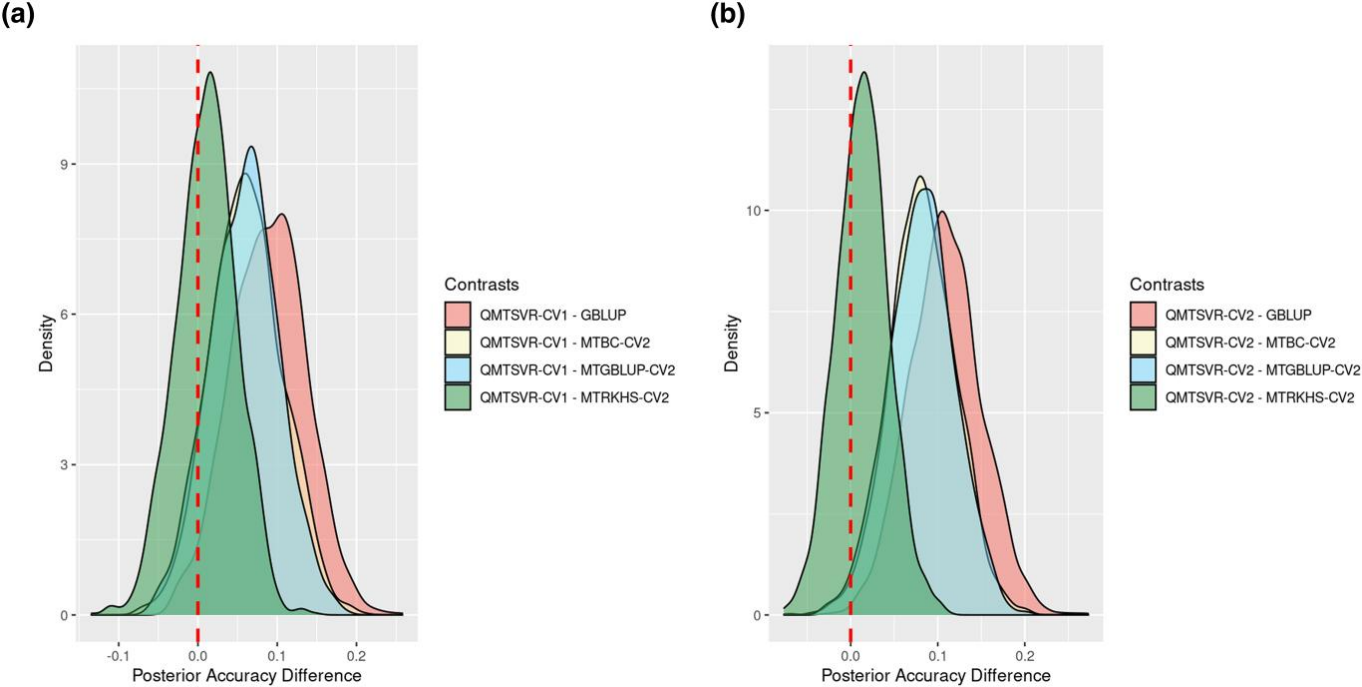
Posterior distribution for the accuracy differences between the model with the highest posterior average for each trait (a and b) and different benchmark models. The accuracy was measured for the de-regressed values of carcass traits (CT1 and CT2) in broiler chickens, according to ST and MT genomic prediction methods. Models were trained using available information for carcass traits until the 19th MG and predictions were obtained for the testing set (from 20th to 23rd MG). For MT models, the available information of secondary traits was analyzed jointly in bi-trait analyses (CT1 × Growth and CT2 × FE). CV1 indicates that the observations for the secondary trait were not available in the testing set. CV2 mimics a scenario where the secondary trait was measured in all animals (including those from the testing set). The accuracy was estimated as the genetic correlation between predicted and observed values multiplied by the square root of heritability for predicted values. These parameters were estimated using only information in the testing set in a bi-trait mixed model analysis. Dashed lines indicate the threshold that suggests no differences in accuracy between models.

We computed the posterior distribution for the $\mathrm{AC}C_{\mathrm{par}}$ differences ($\lambda_{\mathrm{ACC}}$) between the model with the highest rank in each trait (based on the average $\;\mathrm{AC}C_{\mathrm{par}\;}$) and some benchmark models (Fig. 9). For *CT1*, there was relatively weak evidence that the difference between QMTSVR-CV1 and GBLUP is negative (*P*[$\lambda_{\mathrm{ACC}}<0\;$] = 0.027), suggesting a better performance in terms of $\;\mathrm{AC}C_{\mathrm{par}\;}$ for QMTSVR-CV1 (Fig. 9a). For the same trait, the posterior distribution for $\lambda_{\mathrm{ACC}}\;$ also provided evidence of higher $\;\mathrm{AC}C_{\mathrm{par}\;}$ for QMTSVR-CV1, when contrasted with MTGBLUP-CV2 (*P*[$\lambda_{\mathrm{ACC}}<0]$ = 0.087) and MTBC-CV2 (*P*[$\lambda_{\mathrm{ACC}}<0]$ = 0.081). Conversely, there was little evidence of a difference in $\;\mathrm{AC}C_{\mathrm{par}\;}$ between QMTSVR-CV1 and MTRKHS-CV2 (*P*[$\lambda_{\mathrm{ACC}}<0]$ = 0.374) (Fig. 9a). Results were similar for *CT2*, in which the posterior distribution for $\lambda_{\mathrm{ACC}}$ suggested strong evidence that the $\;\mathrm{AC}C_{\mathrm{par}\;}$ assessed for QMTSVR-CV2 was higher than those obtained by GBLUP (*P*[$\lambda_{\mathrm{ACC}}<0]$ = 0.0006), MTGBLUP-CV2 (*P*[$\lambda_{\mathrm{ACC}}<0]$ = 0.013), and MTBC-CV2 (*P*[$\lambda_{\mathrm{ACC}}<0]$ = 0.011; Fig. 9b). Once again, no clear differences in $\;\mathrm{AC}C_{\mathrm{par}\;}$ were found between QMTSVR and MTRKHS when trained under the same CV2 validation design (*P*[$\lambda_{\mathrm{ACC}}<0]$ = 0.319) (Fig. 9b). Moreover, for *CT2*, there was some evidence in favor of a better performance for QMTSVR-CV2 compared to that obtained by the SVR (*P*[$\lambda_{\mathrm{ACC}}<0]$ = 0.137). On the other hand, one cannot be very confident that a real difference exists in $\;\mathrm{AC}C_{\mathrm{par}\;}$ between QMTSVR-CV2 and SVR (*P*[$\lambda_{\mathrm{ACC}}<0]$ = 0.47) (data not shown).

### Computational time

The computational time for running the 20,000 Gibbs samples (averaged over different traits and training strategies) in the multivariate kernel regression models (MTGBLUP and MTRKHS) was 43.11 ± 0.19 (min). Conversely, the computational time for the MTBC model was much larger, taking on average 262.75 ± 0.16 min to run the same number of iterations. The computational time required for the GA-based optimization process for the QMTSVR method was somewhat in the middle, needing on average 78.34 ± 8.99 min. This was the time necessary for building the EDMs based on the marker matrix and running the GA over 25 hyperparameter combinations (executing a multicore parallelized process) during 30 generations, totalizing 750 specific models assessed for each trait and training strategy. For the weighted SVR, there is an additional running time for computing the EDM weighting factors, which will vary depending on the method used.

## Discussion

Since early developments by Henderson and Quaas (1976), MT genetic analysis has become a standard methodology for the joint evaluation of many traits. This approach has a solid theoretical base for pedigree-based selection and is becoming increasingly popular for GS, with different methods and strategies proposed (Calus and Veerkamp 2011; Burgueño *et al*. 2012; Guo *et al*. 2014; Cheng *et al*. 2018; Karaman *et al*. 2018, Lado *et al*. 2018, Gianola and Fernando 2020; Manzanilla-Pech *et al*. 2020). The MTGBLUP is a natural choice for genomic-enabled MT analyses as it can be extended straightforwardly from the ST case, based on well-established mixed model theory (Henderson and Quaas 1976). Nonetheless, models assuming different priors for variable selection or differential shrinkage also exist (Cheng *et al*. 2018; Gianola and Fernando 2020). Here, we presented a simplified approach for expanding the SVR to the MT case under a genomic prediction context. This model shares some computational advantages of the GBLUP (e.g. both are based on *N × N* kernels) while being capable of exploring nonlinear relationships within and between traits, without any strong assumption for the markers’ effects. Results showed that the proposed method was competitive with conventional MT Bayesian regression models using either Gaussian (i.e. MTGBLUP and MTRKHS) or Gaussian–spike–slab (MTBC) multivariate priors.

We used deregressed EBVs (DRP) as target variables, which can be considered as smoothed data points with the influence of parent average effects removed (Garrick *et al*. 2009). Overall, our results indicate that the nonlinear genome-wide prediction methods achieved higher predictive performance for the DRP of both traits (*CT1* and *CT2*), compared to linear models that assumed only additive effects for the marker effects. Breeding values are represented as linear combinations of allelic substitution effects. Accounting solely for additive inheritance attributed to marker effects generally performs better when this assumption holds (Momen *et al*. 2018). However, nonadditive gene action such as epistasis also translates into additive variance when allelic frequencies are low (Hill *et al*. 2008; Huang and Mackay 2016), which is expected for complex traits under directional selection. Accordingly, the additive variance captured from resemblance among relatives may also include a fraction of the variance generated by interaction effects (Huang and Mackay 2016). This might partially explain the occasional superiority of some nonlinear methods over additive models for specific traits and populations (Moser *et al*. 2009; Azodi *et al*. 2019; Abdollahi-Arpanahi *et al*. 2020; Alves *et al*. 2021). Moreover, imperfect linkage disequilibrium between markers and causal mutations can generate “phantom epistasis” (de los Campos *et al*. 2019). Despite its adverse inferential consequences, under some circumstances, this statistical phenomenon may explain the better predictive ability of nonlinear prediction machines even when the target variable is expected to depend on additive effects only (Schrauf *et al*. 2020).

As discussed in previous studies (Burgueño *et al*. 2012; Lado *et al*. 2018), we assessed the predictive performance of MT models under 2 validation designs (CV1 and CV2). CV1 enables the borrowing of information between relatives within and across traits; however, in this scheme, all traits are unavailable for individuals in the testing set. In practice, that means that in CV1, secondary traits do not provide direct information for the prediction of focal traits within the same individual. On the other hand, CV2 reflects a scenario where the target individuals have been evaluated for secondary traits, for example traits that are cheaper to measure and/or expressed earlier in life. In this validation scheme, predictions for the focal trait in the testing individuals borrow information from relatives measured for all traits (focal and secondary traits) as well as from secondary traits measured in the target individuals themselves. This is a reasonable scenario in practice since broiler chickens are selected before slaughtering, i.e. after some routinely measured traits are already available, especially *Growth*-related.

As observed in other studies (Aroujju *et al.* 2020; Shahi *et al*. 2022), the MT methods generally did not present a better performance than ST did when observations for the correlated indicator trait were omitted in the testing set (CV1). This might be due to the low genetic correlations among the traits analyzed in this study. In general, MT methods for genomic prediction are expected to be advantageous when traits are highly correlated and heritability values for the indicator traits are higher than for the target trait (Calus and Veerkamp 2011; Guo *et al*. 2014). In CV2, however, we observed a slight but consistent advantage (higher ACC, smaller RMSE*, and *b* coefficient closer to 1) for the MT methods compared to their respective ST versions in both traits, especially for QMTSVR and MTRKHS. This is in line with Calus and Veerkamp (2011), who found in simulation studies an increase in accuracy of up to 0.04 for animals with only phenotypes on a correlated trait presenting a genetic correlation of 0.25 with the trait of interest.

Nonetheless, because both target and secondary traits are measured on the same individual, predictions for the target trait using a CV2 design can be contaminated by nongenetic factors shared between traits, leading to a possible bias in the estimate of accuracy based on Pearson's correlation (ACC). The extent of this bias will depend on the magnitude and direction of residual covariances between the focal and secondary traits (Runcie and Cheng 2019). For this reason, as suggested in previous works (Runcie and Cheng 2019; Lopez-Cruz *et al*. 2020), we also computed a parametric estimate of accuracy ($\mathrm{AC}C_{\mathrm{par}\;}$) based on the selection index theory. This requires estimating the genetic correlations between predicted and observed values for the target trait in the testing set, as well as the heritability of the predicted values (Lopez-Cruz *et al*. 2020). Interestingly, we observed that model/validation design selection was sensitive to the chosen accuracy metric (ACC or ACC_par_) mostly for *CT1*, which presented a higher residual correlation with *Growth* than that estimated between *CT2* and *FE*. Still, both metrics seem to provide pieces of evidence for better predictive accuracy for the proposed method (QMTSVR) compared to conventional prediction methods such as GBLUP, BC, MTGBLUP, and MTBC, besides a similar performance between QMTSVR and MTRKHS (Figs. 6, 8, and 9). Furthermore, both metrics seem to suggest a higher predictive accuracy for QMTSVR-CV2 over the regular SVR for CT2, although this result did not replicate across accuracy metrics for *CT1*. We believe the main reasons for this limited superiority observed were as follows: (1) the target traits have high heritability and therefore prediction accuracy is already satisfactory for ST models and (2) genetic correlations between target and secondary traits are weak. We expect that the advantage of the proposed method over the univariate case will become clearer in more favorable scenarios (as so for the other conventional MT models).

While the findings of this study suggest that QMTSVR outperformed MTGBLUP and MTBC, it is important to note that this superiority may not hold for other traits and species. As animal and plant breeding lacks a universally best genome-enabled prediction method (Gianola 2021), it is crucial to carefully evaluate and select the appropriate method based on the specific context and objectives. Moreover, the predictive performance of different methods depends on several factors such as trait genetic architecture, linkage disequilibrium extent between markers, training sample size, and genetic connectedness between the training and target samples (Calus 2010; Wang *et al*. 2017; Zhang *et al*. 2019).

Despite the plethora of existing methods, kernel-based methods as genome-assisted prediction machines for complex traits have become popular in animal and plant breeding over recent years, mostly due to the attractive feature of higher flexibility for accommodating hidden nonlinear relationships such as epistasis and genome–environment interactions (Gianola *et al*. 2006; Gianola and van Kaam 2008; Morota and Gianola 2014; Montesinos-López *et al*. 2021). Recently, Montesinos-López *et al*. (2022) reported a Bayesian RKHS for MT genomic prediction. These authors found that the MT-expanded RKHS coupled with a nonlinear Gaussian kernel outperformed the predictive ability of MTGBLUP within and across environments for wheat production traits (Montesinos-López *et al*. 2022). The RKHS method has also been extended to accommodate information from both genotyped and nongenotyped animals using a similar strategy to the widely used single-step GBLUP (Momen *et al*. 2021).

A key difference between the QMTSVR and the kernel ridge regression methods (e.g. GBLUP, RKHS, and their MT counterparts) lies in the loss function used to solve the regularized optimization problem. As for SVR and SVR_Lin, the QMTSVR uses a *ɛ*-insensitive loss, which ignores errors smaller than a constant *ɛ* and assigns an L1 norm to penalize large errors above or below this threshold, and all the remaining kernel methods use the common squared loss function coupled with an L2 regularization parameter. The adoption of the *ɛ*-insensitive loss function provides sparse solutions since only a subset of training data points that lie outside the insensitive margin (i.e. the SVs) receive nonzero regression coefficients. Both QMTSVR and MTRKHS are potentially able to map nonlinear relationships by using nonlinear kernels. A major difference between these 2 methods is that the implemented MTRKHS uses the regular scaled RBF kernel, which needs a single bandwidth hyperparameter to control the kernel variance. On the other hand, the QMTSVR uses a MT scaled RBF kernel that assigns t(t+1)/2 different values for the kernel bandwidth, which means that the control of the kernel variance is specific to trait combinations.

Moreover, in the QMTSVR, the genetic and residual covariance components do not need to be estimated for incorporating MT information in genome-enabled prediction. Our approach might be useful for predicting novel traits with small training sizes based on the additional information provided by correlated traits. However, there is a lack of interpretability in terms of effects captured by the QTMSVR. It is important to note that QMTSVR-predicted values are not construed as estimated breeding values since conditioning predictions on indicator traits alter the variance component accordingly. Hence, predicted values must be interpreted on the scale of the training variables rather than as DGVs. Additionally, ML methods generally need several hyperparameters to be tuned during training, which can be computationally intensive. Here, the burden of fine-tuning was alleviated by adapting some global hyperparameters for all traits, hence reducing the space of possible combinations.

A GA was then employed for avoiding an extensive grid search in the hyperparameter space. GA is a metaheuristic approach used to solve nonconvex optimization problems in discrete and continuous systems. The GA uses a population of chromosomes (generally represented by binary arrays for encoding the real problem in the algorithm language) for exploring the search space in different directions simultaneously. The main features of this algorithm are inspired by natural selection, i.e. the fittest individuals (those with higher or lower scores for the cost function, depending on the problem) have higher chances to generate new chromosomes for the next generation. Although GA does not guarantee global optima for the solutions, this nonlinear optimization procedure is very appealing for ML since it is flexible enough to address different complex problems (e.g. feature selection, hyperparameter tuning, and solving constrained systems) and can be easily parallelized. Results showed that finding an appropriate hyperparameter configuration with GA for QMTSVR would considerably take less computing time than fitting a Bayesian regression model with multivariate spike–slab priors. The multivariate Bayesian kernel regression models (MTGBLUP and MTRKHS) were the most efficient in terms of computational time. Nevertheless, one must consider that for MTRKHS, the time needed to fine-tune the bandwidth hyperparameter (generally through grid search) was ignored, as we set the same value as used for SVR (optimized via GA). Notice that comparisons performed here are only valid for the number of iterations used for the GA and Gibbs sampling algorithms.

Similar to what has been proposed for GBLUP (Karaman *et al*. 2018; Gianola *et al*. 2020), we expanded the QMTSVR to account for the relative importance of different genomic regions, by computing trait-specific and pleiotropy-weighted kernels. We found that this strategy did not improve predictive ability compared to the nonweighted counterpart. This might be due to the weak correlations observed between traits. One must highlight that the weighting factors we used were based on SNP effects estimated from a multivariate multiple regression. The preceding approach is simplistic and may not add much relevant information on the architecture of the traits. Future work using weighting factors derived from MT Bayesian regression models or other ML methods such as random forest and gradient boosting may provide better results.

In summary, we introduced a fully nonparametric and nonlinear procedure for integrating MT information into genomic prediction tasks. Preliminary results suggested that QMTSVR has the potential to achieve a competitive or even better performance than the compared multivariate Bayesian shrinkage and variable selection models. The main advantages of QMTSVR are its flexibility for capturing extra variance due to nonlinear effects and the unnecessity of estimating covariance parameters, which generally require a large amount of data and may hamper the joint analysis of many traits with small training samples. This scenario is very common for novel traits that are hard or expensive to measure. QMTSVR uses the standard SMO algorithm for solving constrained optimization equations, which does not require the repeated inversion of dense matrices. Furthermore, the fine-tuning process is performed internally using a GA with a multithread parallelization process.

## Conclusion

We assessed strategies for extending the SVR method to a MT-GP context. Results showed that the proposed approach is competitive with conventional MT Bayesian regression models using either Gaussian or spike–slab multivariate priors. QMTSVR represents a nonparametric alternative to MT-GP, and it is capable of handling nonadditive effects without adding much computation complexity. Future research performing a comprehensive comparison of the proposed approach with other methods and different traits is encouraged. Genomic prediction of postmortem carcass traits in broilers can be improved when correlated secondary traits measured in vivo are available for target individuals. Nonetheless, differences in accuracy observed between ST and MT models were small, possibly because target traits investigated in this study were highly heritable and presented weak genetic correlations with the indicator traits.

## Supplementary Material

jkad109_Supplementary_Data

## Data Availability

All data supporting the results and conclusions of this study are fully described within the manuscript and in the supplementary files. Phenotypes and kernels necessary to reproduce the genomic prediction analyses can be found at the following link: https://doi.org/10.6084/m9.figshare.21538350. The source code for the developing version of the *qmtsvr* package can be found at: https://github.com/alvesand/qmtsvr. Supplemental material available at G3 online.

## Appendix A

This section provides further details on the connection between the primal and dual forms of the standard SVR algorithm. A complete theoretical description is beyond the scope of this article; the interested reader can refer to Vapnik *et al*. (1996) and Smola and Schölkopf (2004) for a comprehensive theoretical foundation.

In its primal form, the SVR general formulation takes the following matrix notation:

(A1)
$$y=b_{0}+\;\phi (M)\beta +e$$

where β is the vector of unknown coefficients, ϕ(M) represents a linear or nonlinear mapping of an *N × p* matrix of predictor variables, $b_{0}$ is the model bias, and **e** is the vector of random errors. Generally, optimal solutions for equation (A1) are found by solving the following convex optimization problem:

(A2)
$$\arg\;\min\;\frac{1}{2}\beta^{T}\beta +\;C(\xi \;+\;\xi^{*}{)}^{T}1_{n},$$

subject to the following inequality constraints:

$$y-\phi (M)\beta -b_{0}\;\leq \;\varepsilon_{n}+\;\xi ,$$

$$\phi (M)\beta +b_{0}-\;y\;\leq \;\varepsilon_{n}+\;\xi^{*},$$

and $\xi_{i},\;\xi_{i}^{*}\;\geq 0,\;\mathrm{for}\;i\;=\;1,2,\ldots ,N$.

The core approach for solving equation (A2) is to build a Lagrangian function from the primal form and the set of inequality constraints (Smola and Scholkopf 2004). Adding a dual set of positive Lagrange multipliers concerning all constraints yields the following dual objective function:

(A3)
$$L_{\beta ,b_{0},\xi ,\xi^{*},\;\alpha ,\alpha^{*},\lambda ,\lambda^{*}}=\;\;\;\frac{1}{2}\beta^{T}\beta +\;C(\xi \;+\;\xi^{*}{)}^{T}1_{n}+\;\;\alpha^{T}(y-\phi (M)\beta -b_{0}-\;\varepsilon_{n}-\;\xi )+\;\alpha^{*T}(\phi (M)\beta +b_{0}-\;y\;-\;\varepsilon_{n}-\;\xi^{*})-(\lambda^{T}\xi +\;\lambda^{*T}\xi^{*}),$$

where $L_{\beta ,b_{0},\xi ,\xi^{*},\;\alpha ,\alpha^{*},\lambda ,\lambda^{*}}$ is the Lagrangian function and $\alpha ,\;\alpha^{*},\;\lambda ,\;\lambda^{*}$ are vectors of positive Lagrange multipliers.

The optimal solutions for $L_{\beta ,b_{0},\xi ,\xi^{*},\;\alpha ,\alpha^{*},\lambda ,\lambda^{*}}\;$ can be found by taking its partial derivatives for the primal variables ($\beta ,\;b_{0},\xi ,\;\xi^{*}$) and setting them to zero:

(A4)
$$\frac{\partial L}{\partial \beta }=0\;\Rightarrow \;\beta -\alpha^{T}\phi (M)\;+\;\alpha^{*T}\phi (M)=0\Rightarrow \;\beta =(\alpha -\alpha^{*}{)}^{T}\phi (M)\;,$$

(A5)
$$\frac{\partial L}{\partial b_{0}}=0\;\Rightarrow \;-\alpha^{T}1_{n}\;+\;\alpha^{*T}1_{n}=0\;\Rightarrow (\alpha -\alpha^{*}{)}^{T}1_{n}=0\;,$$

(A6)
$$\frac{\partial L}{\partial \xi }=0\;\Rightarrow \;\;C1_{n}^{T}1_{n}-\alpha^{T}1_{n}-\;\lambda^{T}\;1_{n}=0\;\Rightarrow \;C1_{n}=\;\alpha +\;\lambda \;,$$

(A7)
$$\frac{\partial L}{\partial \xi^{*}}=0\;\Rightarrow \;\;C1_{n}^{T}1_{n}-\alpha^{*T}1_{n}-\;\lambda^{*T}\;1_{n}=0\;\Rightarrow \;C1_{n}=\;\alpha^{*}+\;\lambda^{*}.$$

Plugging back equations (A4–A7) into equation (3) yields the dual optimization problem:

(A8)
$$\arg\;\max\;-\frac{1}{2}(\alpha -\alpha^{*}{)}^{T}\phi (M)\phi (M{)}^{T}(\alpha -\alpha^{*})-\;(\alpha +\alpha^{*}{)}^{T}\varepsilon_{n}+(\alpha -\alpha^{*}{)}^{T}y$$

$$\mathrm{subject}\;\mathrm{to}\;\{\begin{array}{c} 0\leq \;\alpha_{i},\;\alpha_{i}^{*}\;\leq C,\;i\;=\;1,2,\ldots ,N\;\;\\ {\left(\alpha -\alpha^{*}\right)}^{T}1_{n}=0 \end{array}.$$

In equation (A8), $\phi (M)\phi (M{)}_{H}^{T}=\;K$ is an *N × N* (semi)positive definite kernel matrix in the Hilbert space. Therefore, using equation (A8) allows us to describe the SVR algorithm only in terms of dot products between the mapped inputs, without explicitly computing ϕ(M) or β.

Using results from equations (4) and (8), the original SVR formulation in equation (1) can be rewritten as:

(A9)
$$y=b_{0}+\;K(\alpha -\alpha^{*})+e,$$

in which **K** must meet the conditions established by Mercer's theorem (Smola and Scholkopf 2004).

Finding an optimal solution for $b_{0}$ can be done by exploring the complementary slackness property of the so-called Karush–Kuhn–Tucker (KKT) conditions, which state that for every inequality constraint, either *f*($x_{i}$) or the corresponding dual variable must vanish for optimal solutions, yielding the following statements (Smola and Scholkopf 2004):

(A10)
$$\begin{array}{c} \alpha_{i}\left(y_{i}-\overset{N}{\underset{\;j=1}{\sum }}\;K(m_{i},\;m_{j})(\alpha_{j}-\alpha_{j}^{*})-b_{0}-\;\epsilon \;-\;\xi_{i}\right)=0\\ \alpha_{i}^{*}\left(\overset{N}{\underset{\;j=1}{\sum }}\;K(m_{i},\;m_{j})(\alpha_{j}-\alpha_{j}^{*})+b_{0}-\;y_{i}\;-\;\epsilon -\;\xi_{i}^{*}\right)=0 \end{array}.$$

One can conclude that data points lying inside the ε-insensitive tube must have null values for $\alpha_{i}$ and $\alpha_{i}^{*}$ to satisfy equation (A10), while observations outside the boundary margin [($f(m_{i})\;+\epsilon -orf(m_{i})\;\text{–}\varepsilon$)] cannot have nonzero values for $\alpha_{i}$ and $\alpha_{i}^{*}$ simultaneously. In the dual form, all training examples that have nonzero $\alpha_{i}$ or $\alpha_{i}^{*}$ are called SVs. Using results from equation (A10), one can recover $b_{0}$ from SVs lying on the hyperplane margin, by noticing that $b_{0i}=y_{i}-\;\overset{\mathrm{NSVs}}{\underset{\;j=1}{\sum }}\;K(m_{i},\;m_{j})(\alpha_{j}-\alpha_{j}^{*})-\;\epsilon$ and $b_{0i}=y_{i}-\;\overset{\mathrm{NSVs}}{\underset{\;j=1}{\sum }}\;K(m_{i},\;m_{j})(\alpha_{j}-\alpha_{j}^{*})+\;\epsilon$ for null values of $\xi_{i}$ and $\xi_{i}^{*}$, respectively.
