# Supplementary material for: (Quasi) multitask support vector regression with heuristic hyperparameter optimization for whole-genome prediction of complex traits: a case study with carcass traits in broilers
Source: G3 (Bethesda). 2023 May 22;13(8):jkad109. doi: 10.1093/g3journal/jkad109 (PMC10411556; doi:10.1093/g3journal/jkad109)
Supplement: jkad109_Supplementary_Data [file jkad109_supplementary_data.pdf]

SUPPLEMENTARY FILES

Algorithm 1: *Genetic Algorithm for Tuning the QMTSVR hyperparameters*

Input: population size (*ps*), number of generations (n_{gen}), mutation rate (*mr*), tournament size (*ts*), crossover rate (*cr*), number of best individuals to keep from previous generations (n_{keep})

Output: The fittest hyperparameter combination in the last generation

```
population = [list with n arrays representing different candidate models];
generation = 0;
while (generation <= n_gen)
  {
   train_and_predict (population);
   return population fitness scores;
   for (i in ps)
     {
       do tournament selection;
       select parent 1 from ts candidates;
       do tournament selection;
       select parent 2 from ts candidates;
       crossover (parent 1, parent 2);
       return child;
       mutate (child); with probability mr for a single bit
       append child to new_population
       train_and_predict (child)
       store child fitness score
     }
   replace n_keep worst individuals of the new_population with n_keep best individuals from the previous generation
   let new_population be population
  }
 return population
 end
```

Figure S1. Genetic algorithm pseudo code for tuning the QMTSVR hyperparameters.

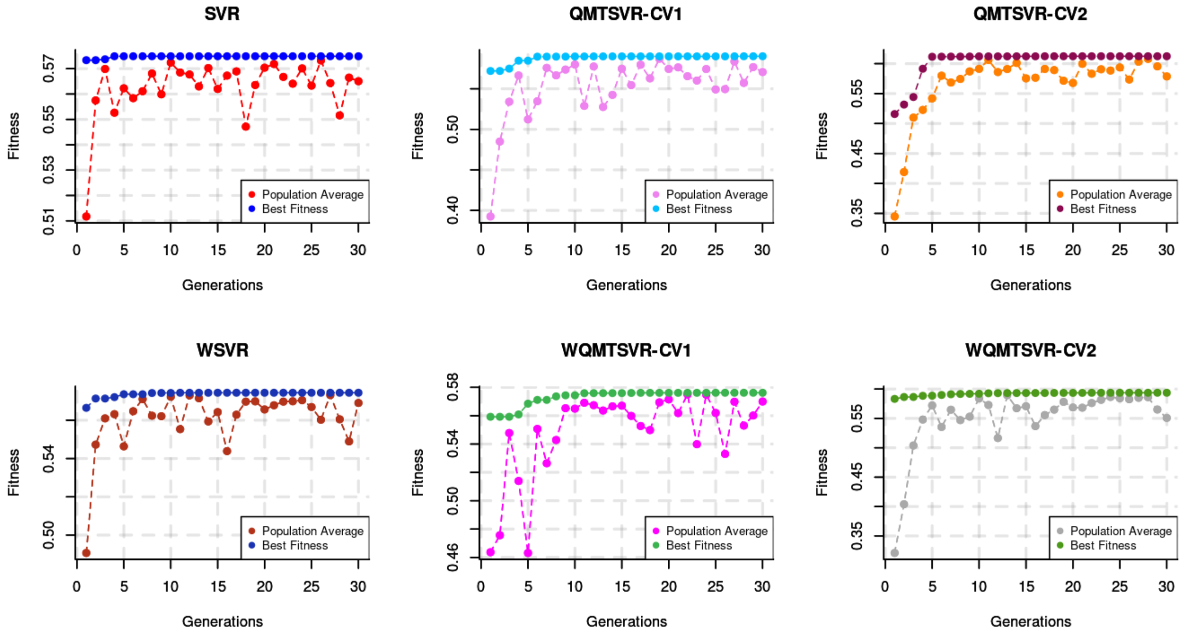

Figure S2. Best and average performance across generations of the genetic algorithm optimization for support vector regression (SVR) models with different hyper-parameters. *Models were trained using available information for carcass trait 1 (CT2) until the 16th mating generation (MG). The vertical axis indicates the performance of selected models for predicting future CT2 observations from the 17th to 19th MG. QMTSVR stands for (quasi) multi-task SVR, in which the information for Feed Efficiency was also included for animals born until the 16th MG (QMTSVR-CV1) or considering that such information was available for all animals, including those from the testing sample (QMTSVR-CV2). WSVR, WQMTSVR-CV1, and WQMTSVR-CV2 are the corresponding models weighted with loci-specific information obtained in genome-wide association studies.*

Table S1. Hyperparameters selected via genetic algorithm for single and multi-task support vector regression models used for genomic prediction of carcass traits in broilers.

Trait	Method[1]	Hyperparameters[2]					
		C	ϵ	θ_{11}	θ_{12}	θ_{22}	ρ_{12}
CT1	SVR	0.2841	0.0001	2.7285	-	-	-
	WSVR	0.4682	0.0001	2.1809	-	-	-
	QMTSVR-CV1	0.3301	0.0001	1.3048	7.0000	6.0143	0.1962
	QMTSVR-CV2	0.4682	0.0001	1.0857	6.8905	3.0571	0.1743
	WQMTSVR-CV1	0.2381	0.0001	2.2905	6.6714	0.1000	0.1086
	WQMTSVR-CV2	0.4682	0.0001	1.8524	7.0000	3.6047	0.1743
CT2	SVR	0.1921	0.0001	3.6048	-	-	-
	WSVR	0.2841	0.0001	1.9619	-	-	-
	QMTSVR-CV1	0.4682	0.0001	2.4000	2.5095	2.1809	0.3057
	QMTSVR-CV2	0.4683	0.0001	2.2905	7.0000	4.9190	0.2071
	WQMTSVR-CV1	0.2381	0.0001	3.6048	3.2762	3.1667	0.0100
	WQMTSVR-CV2	0.3762	0.0001	3.6048	3.3857	2.0714	0.1414

[1]Methods were trained using the available information for carcass trait 1 (CT1) or carcass trait 2 (CT2) until the 16th mating generation (MG). The genetic algorithm optimized the models for predicting future observations from the 17th to 19th generations. SVR stands for support vector regression (SVR) using only the observations of the target variable (CT1 or CT2) as the training sample. QMTSVR stands for (quasi) multi-task SVR, in which the information for an indicator trait was also included for animals born until the 16th MG (QMTSVR-CV1) or considering that such information was available for all animals, including those born in the testing sample (QMTSVR-CV2). WSVR, WQMTSVR-CV1, and WQMTSVR-CV2 are the equivalent models weighted with loci-specific information obtained in genome-wide association studies. [2]C: regularization parameter; ϵ: the epsilon parameter for the ϵ-insensitive loss function; θ_{11}: the bandwidth for the radial basis kernel of the target trait; θ_{12}: a bandwidth hyperparameter for the kernel linking the observations of both target and indicator traits; θ_{22}: the bandwidth hyperparameter for the kernel of the indicator trait; ρ_{12}: a weighting constant for the linking kernel.
